# Essential Oils of *Melaleuca, Citrus*, *Cupressus*, and *Litsea* for the Management of Infections Caused by *Candida* Species: A Systematic Review

**DOI:** 10.3390/pharmaceutics13101700

**Published:** 2021-10-15

**Authors:** Rafael Alves da Silva, Flávia Maria Pinto Monteiro Antonieti, Denise Von Dolinger de Brito Röder, Reginaldo dos Santos Pedroso

**Affiliations:** 1 Post-Graduation Program in Health Sciences, Federal University of Uberlândia, Uberlândia 38400-902, MG, Brazil; allvesraf@gmail.com (R.A.d.S.); flaviamantonieti@gmail.com (F.M.P.M.A.); 2 Institute of Biomedical Sciences, Federal University of Uberlândia, Uberlândia 38400-902, MG, Brazil; 3 Technical School of Health, Federal University of Uberlândia, Uberlândia 38400-902, MG, Brazil; rpedroso@ufu.br

**Keywords:** essential oils, anti-*Candida* activity, alternative therapy

## Abstract

*Candida* is a common agent of infection in humans, which has a wide distribution and is a colonizer fungus of the body, occasionally assuming the role of a pathogen. The type of treatment depends on the site of infection and the clinical condition of the patient. Superficial infections, such as mucosal infections, can be treated with topical medications. So-called alternative therapies have rarely been studied, although the literature records the effectiveness of some treatments, especially as complementary therapy. The aims of this review were to analyze evidence of the anti-*Candida* inhibitory activity of essential oils of the *Citrus*, *Cupressus*, *Litsea*, and *Melaleuca* species; in addition to addressing the chemical composition, probable mechanisms of antifungal action and studies of toxicity, cytotoxicity, and genotoxicity were included. The literature from Medline/PubMed, Science Direct, Scopus, Web of Science, and the Brazilian database Periodic Capes was reviewed. Thirty-eight articles were selected, which included two articles on *Litsea* spp., seven on *Cupressus* spp., thirteen articles on *Citrus* spp., and twenty-one articles on *Melaleuca* spp. In conclusion, this study showed in vitro evidence for the use of essential oils of the plant species evaluated for the treatment of infections caused by different *Candida* species.

## 1. Introduction

Mycoses caused by *Candida* species are the most frequent opportunistic fungal infections affecting humans. The clinical manifestations are the most varied, from superficial and subcutaneous to deep and disseminated infections [1]. More serious infections occur in hospitalized patients, who are often immunocompromised, undergoing invasive procedures, or using antibacterial drugs [2]. The most frequent species include *C. albicans* and others, often referred to as non-*C. albicans* species, such as *C. tropicalis, C. parapsilosis, C. glabrata*, and *C. krusei* [3].

Non-invasive infections include those that affect the oral cavity, vagina, penis, and other parts of the body. Oral candidiasis is the most common, affecting the oral mucosa, tongue, and throat, followed by vulvovaginal candidiasis, causing vaginal discharge and other signs and symptoms. Penile infection, on the other hand, is less frequent [1].

*Candida* species resistance to some antifungal agents has been known for decades (for example, the intrinsic or acquired resistance, respectively, of *C. krusei* and *C. glabrata* to fluconazole). This resistance increases the need for new alternative treatment proposals [4]. Currently, the emergence of *C. auris*, a *Candida* species that has shown resistance to most of the available antifungal drugs, has aroused interest in the search for new therapeutic alternatives [5].

The search for new drugs with an antifungal effect, a wider spectrum, or different from the existing ones can minimize the impact of the dissemination of resistant isolates. Natural products, including those obtained from plants, have shown a considerable diversity of chemical constituents that have in vitro antimicrobial activity, with potential for clinical use [6,7,8].

Essential oils (EOs) include natural products obtained from plants that are widely used in the industry and have potential as agents with antimicrobial activity, meaning that they can be explored for the treatment of human and animal infections. Antimicrobial activity is often attributed to the association of major components present in EOs [9,10]. The proposed mechanisms of action are diverse, including a direct action on the microbial cell, the interaction with the host’s immune system, and others. These general mechanisms try to define which chemical components are responsible for the antifungal effect [8].

According to previous studies, the EOs of species of the *Litsea, Citrus*, and *Cupressus* have anti-*Candida* effects in vitro [11]. In addition, *Melaleuca alternifolia* has been reported by its antimicrobial activity for a long time [7,12,13,14,15,16]. All of the EOs are interesting options for the alternative and complementary treatment of clinically relevant microorganisms, such as *Candida* species, the main cause of superficial mycoses in humans.

In this way, the aim of this review was to analyze evidence of the anti-*Candida* inhibitory effect of essential oils from the species of *Citrus, Cupressus, Litsea*, and *Melaleuca*, in addition to addressing the phytochemical composition, possible mechanisms of antifungal action and toxicity, cytotoxicity, and genotoxicity studies.

## 2. Materials and Methods

### 2.1. Study Design

This review was carried out through a systematic literature search addressing the anti-*Candida* inhibitory effect of essential oils from *Citrus, Cupressus, Litsea*, and *Melaleuca*. The research was registered in PROSPERO (No. CRD42020188918). Independently, the assessment of the risk of bias for each included article was performed by two reviewers, and disagreements were resolved by discussing until reaching a consensus with a third reviewer.

### 2.2. Search Strategy

The research was conducted in the Medline/PubMed, Science Direct, Scopus, Web of Science, and Brazilian database Periodic Capes using the terms (“anticandidal” OR “antifungal”) AND (“fungal” OR “*Candida*”) AND (“volatile oil” OR “essential oil”) AND (“mycoses” OR “candidiasis” OR “infections”) AND (“*Citrus*” OR “*Melaleuca*” OR “*Cupressus*” OR “*Litsea*”). For Science Direct: (“anticandidal” OR “antifungal”) AND (“fungal” OR “*Candida*”) AND (“volatile oil” OR “essential oil”) AND (“candidiasis” OR “infections”) AND (“*Citrus*” OR “*Melaleuca*” OR “*Cupressus*” OR “*Litsea*”) and (“anticandidal” OR “antifungal”) AND (“fungal” OR “*Candida*”) AND (“volatile oil” OR “essential oil”) AND (“mycoses” “ OR “infections”) AND (“*Citrus*” OR “*Melaleuca*” OR “*Cupressus*” OR “*Litsea*”).

### 2.3. Selection of Articles, Inclusion, and Exclusion Criteria

The publications considered for inclusion in this review were those published from 2011 to 2020, containing the following information: (I) Biological activity: antifungal activity involving *Candida* species; (II) Plants and derivatives: essential oils only; and (III) Study design: Experimental in vitro, laboratory studies using the broth dilution assay (CLSI—Clinical and Laboratory Standards Institute and EUCAST—The European Committee on Antimicrobial Susceptibility Testing, and adaptations), agar diffusion disk (Kirby–Bauer and adaptations), and agar dilution assay, preclinical studies, case reports, randomized clinical trials, cross-sectional studies, and prospective studies. The exclusion criteria were the lack of access to the full content of the published article.

### 2.4. Study Analysis

The information collected in the articles was descriptively evaluated and grouped according to the essential oil (EO), *Candida* genus and/or species, chemical constitution, minimum inhibitory concentration (MIC), minimum fungicidal concentration (MFC), minimal biofilm inhibitory concentration (MBIC), and minimal biofilm eradication concentration (MBEC), in addition to information on synergism with antifungals or EOs. Experimental toxicity in vitro and in vivo, such as mean inhibitory concentration (IC_50_), mean lethal concentration (LC_50_), mean lethal dose (LD_50_), and genotoxicity, was also evaluated.

## 3. Results

### 3.1. Characteristics of the Studies

**Figure 1:**
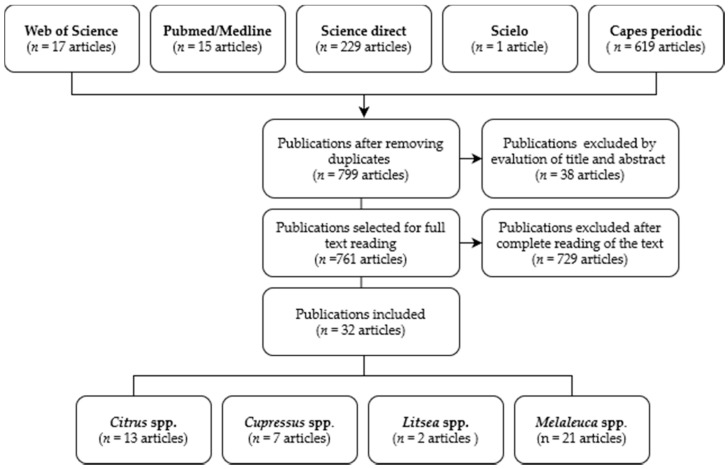
Flow diagram of retrieved, selected, included, and excluded studies.

The search of databases identified 881 studies; after analysis according to the inclusion and exclusion criteria, 32 publications were eligible, including two articles on *Litsea* spp., seven on *Cupressus* spp., thirteen articles on *Citrus* spp., and twenty-one articles on *Melaleuca* spp. Figure 1 shows the flow of articles included in this study.

Most studies related the in vitro antifungal effect against *Candida* species isolates and used different methodologies and techniques, with many adaptations and variations from those recommended by the CLSI and EUCAST, and based on other research. Accordingly, in vitro studies showed antifungal susceptibility testing by macro- and microdilution methodologies in broth (with results expressed in μg/mL, μL/mL, and in percent—% *v*/*v*), which exhibit the minimum inhibitory concentration, and still, some others reported the minimum fungicidal concentration. In addition, other studies used the agar diffusion methodology from the well or the disk (results expressed in mm). Studies that addressed other techniques or technologies, or even activities on biofilm, are reported and discussed in the text and shown in Tables and in Supplementary Materials. A list of *Melaleuca* spp., *Citrus* spp., *Cupressus* spp., and *Litsea* spp. essential oils and their main components related in the text, as well as a summary of the most important results, are provided in the Supplementary Materials.

The origin of EO was also the cause of the differences observed between the susceptibilities of different isolates and species, in addition to other variations observed between different studies.

### 3.2. Melaleuca spp.

The genus *Melaleuca* includes plants belonging to the Myrtaceae family, such as the species *M. linariifolia*, *M. viridiflora*, *M. dissitiflora*, M*. leucadendra*, *M. acacioides*, *M. ericifolia*, *M. alsophila*, and *M. alternifolia* [17,18,19]. Among these species, the EO of *M. alternifolia*, also known as Tea tree, is the most widespread globally. The product of the distillation of the leaves and branches of *M. alternifolia* is traditionally known by native peoples of Oceania for its anti-infective and anti-inflammatory properties in medicinal preparations [19]. Tea tree EO is used in topical antimicrobial formulations in the pharmaceutical and cosmetic industries and has been used as a flavoring in the food industry [12,17,18,19,20].

#### 3.2.1. Chemical Composition of Essential Oils from *Melaleuca* spp.

Tea tree EO presents a diversity of constituents (Supplementary Materials), which varies according to the origin and region in which the plant was cultivated [17]. The constituent variation is even greater when different species of *Melaleuca* are considered [20,21,22].

The main components described in the EO of *M. alternifolia* leaves are terpinen-4-ol (35.93–47.5%), *γ*-terpinene (17.8–23.58%), α-terpinene (6.84–11.91%), and limonene (1–19.79%) [13,14,22,23].

In the leaves, bark, fruits, and in tips of branches of *M. leucadendra*, the following were identified: α-eudesmol (13.7–30.7%), guaiol (7.3–12.5%), (E)-caryophyllene (3.8–7%), 1,8-cineole (0.2–5.2%), linalool (1.4–5.1%), and bulnesol (2.2–5.3%) [15]. The analysis of the species *M. quinquenervia* revealed 1,8-cineol (40.3%), carveol (27.15%), and myrtenol (9.43%) to be the most frequent constituents [21].

#### 3.2.2. Anti-*Candida* Activity of *Melaleuca* spp. Essential Oil

Tea tree EO has shown antifungal effects in vitro against several *Candida* species, with suitable efficacy when used topically in the treatment of oral candidiasis [24]. The EO of *Melaleuca* at a concentration of 0.5% (*v*/*v*) (equal to MIC_50_) acts by inhibiting both the initial adhesion and the subsequent stages of *C. albicans* biofilm formation [24]. The mechanism of action is associated with changes in the structure of the fungus cell membrane, making it permeable [12] (Table 1).

The in vitro antifungal effect has been described in different studies, regardless of the methodology and technique used, whether by disk diffusion in agar, macrodilution and microdilution in broth, or agar dilution. This was demonstrated in studies using the EO of *M. alternifolia*, showing inhibitory effects against different species of *Candida* spp. [7,25,26]. By disk diffusion in agar, the EO of *M. alternifolia* showed inhibitory effects against *C. albicans* [14,22] and also for the species *C. kefyr, C. dublinensis, C. lusitaniae*, and *C. parapsilosis* [20] (Table 1).

*C. albicans* is the most studied species, possibly because it is the species most related to human infections. The MIC for the EO of *M. alternifolia*, expressed in % (*v*/*v*), varies according to the study, with values as low as 0.12–4% (*v*/*v*) [7,15,23,25,26,27]. Moreover, other studies reported an MIC (*m*/*v*) of 0.0097–5 mg/mL (which is the same as 9.7–5000 μg/mL) [8,11,14,21] (Table 1).

In *C. albicans,* the isolates susceptible and resistant in vitro to fluconazole are evaluated [7] with an MIC reported to range from 0.06–0.5% (*v*/*v*), and 5–20 mg/mL (equivalent to 5000–20,000 μg/mL), respectively, and no differences between the MICs of the two groups of isolates were found [13] (Table 1).

The studies that included the evaluation of tea tree EO activity on *C. glabrata* also showed an in vitro inhibitory effect. Other MIC values range from 0.156–4% (expressed in *v*/*v*) [4,16,27,28], and have also been seen in the range of 9.7–625 μg/mL (equivalent to 0.0097–0.625 mg/mL) [14]. On the other hand, values greater than 2000 μg/mL have also been reported [11].

For *C. parapsilosis*, MICs greater than 2000 μg/mL [11] and 5 mg/mL [14] (equivalent to 5000 μg/mL) have been reported. Other studies have found results ranging from 0.312–1% (*v*/*v*) for *C. krusei* isolates [4,27,29] (Table 1).

An isolate of *C. tropicalis* resistant to nystatin, fluconazole, and voriconazole in vitro presented an MIC of 8% (*v*/*v*) [27]. Other studies found MICs equal to 1 mg/mL (1000 μg/mL) [21], while MIC values were found up to the limit of 2000 μg/mL, which was the highest concentration used in the study [11] (Table 1).

Other *Candida* species have also shown inhibition by EO of *Melaleuca*, as demonstrated for the species *C. boidinni*, *C. colliculosa, C. dubliniensis, C. famata, C. lusitaniae, C. pelliculosa,* and *C. rugosa* [14,27], with an MIC less than or equal to 1% (*v*/*v*), and including isolates resistant to the drugs nystatin, fluconazole, and voriconazole [27] (Table 1).

The EO of *M. alternifolia* shows inhibitory activity against biofilm formation of *C. albicans* [24,26]. For this species, no concentration capable of inhibiting biofilm formation was observed within the limits of those tested (up to 8% *v*/*v*), *C. albicans* isolates, whose MIC was 0.2% (*v*/*v*) [26]. On the other hand, the concentration of 1% EO (*v*/*v*) was able to inhibit the development of biofilm in cases where the MIC was 0.5% (*v*/*v*) [24], while 12.5% (*v*/*v*) could eradicate biofilms formed by *C. albicans* [15]. The EO of *M. alternifolia*, when incorporated into nanoparticles, showed a greater antibiofilm inhibitory effect in vitro when compared to the EO at a concentration of 15.6%, which was able to inhibit more than 70% of the biofilm formed by *C. glabrata* [30] (Table 1).

Other species of *Melaleuca* have also been evaluated for their in vitro inhibitory activity against *C. albicans*. The EO of *M. quinquenervia* had an MIC of 4 mg/mL (equivalent to 4000 μg/mL) [21], and the EO of *M. leucadendra*, extracted from the bark, leaves, and fruits, had an MIC of 64, 128, and 256 μg/mL, respectively [20] (Table 1).

In vivo antifungal activity after the use of the EO *of M. alternifolia* showed a reduction of colonization by *C. albicans* in the oral cavity in different sensitivity profiles [13,15]. An in vivo study in mice showed that 4% (*v*/*v*) EO had a protective action after two days of treatment against oral candidiasis induced by *C. albicans* isolates that were susceptible and resistant to fluconazole [13], while there was still the presence of tissue lesions characteristic of candidiasis in the oral cavity tissue of mice after 24 h of treatment, even when treated with 12.5% (*v*/*v*) [15].

**Table 1:** In vitro antifungal activities of *Melaleuca* spp. essential oils tested against *Candida* species according to different methods.

*Melaleuca* Species	Method of Antifungal Susceptibility	Species of *Candida* (Number of Strains Tested)	Agar Diffusion * or MIC **	Reference
*M. alternifolia*	Disk diffusion	*C. albicans* (19)	12–25 mm	[14,22]
		*C. atlântica* (1)	21.1 mm	[14]
		*C. dublinenensis* (1)	15 mm	[14]
		*C. famata* (1)	20.66 mm	[14]
		*C. glabrata* (3)	11.66–14.33 mm	[14]
		*C. intermedia* (1)	20 mm	[14]
		*C. kefyr* (2)	19.33–25.3 mm	[14]
		*C. lusitaniae* (1)	15.33 mm	[14]
		*C. marítima* (1)	24.66 mm	[14]
		*C. parapsilosis* (1)	14.66 mm	[14]
		*C. sake* (1)	16.33 mm	[14]
*M. alternifolia*	Broth microdilution	*C. albicans* (207)	0.125–4% (*v*/*v*)	[4,7,15,23,24,26,27]
		*C. boidinii* (3)	0.12–0.25% (*v*/*v*)	[27]
		*C. colliculosa* (1)	0.25% (*v*/*v*)	[27]
		*C. famata* (2)	0.25–0.5% (*v*/*v*)	[27]
		*C. glabrata* (52)	0.156–4% (*v*/*v*)	[4,16,27,28]
		*C. krusei* (13)	0.12–0.625% (*v*/*v*)	[4,27,29]
		*C. lusitaniae* (5)	0.25–1.0% (*v*/*v*)	[27]
		*C. pelliculosa* (1)	0.5% (*v*/*v*)	[27]
		*C. rugosa* (1)	0.12% (*v*/*v*)	[27]
		*C. tropicalis* (1)	8% (*v*/*v*)	[27]
*M. alternifolia*	Broth microdilution	*C. albicans* (20)	0.0097–20 mg/mL	[13,14,22]
		*C. atlântica* (1)	0.0097 mg/mL	[14]
		*C. dublinenensis* (1)	0.0195 mg/mL	[14]
		*C. famata* (1)	0.0097 mg/mL	[14]
		*C. glabrata* (3)	0.0097–0.625 mg/mL	[14]
		*C. intermedia* (1)	0.0097 mg/mL	[14]
		*C. kefyr* (2)	0.0097 mg/mL	[14]
		*C. lusitaniae* (1)	0.0097 mg/mL	[14]
		*C. maritima* (1)	0.0097 mg/mL	[14]
		*C. parapsilosis* (1)	5 mg/mL	[14]
		*C. sake* (1)	0.0097 mg/mL	[14]
*M. alternifolia*	Broth microdilution	*C. albicans* (2)	625 to >2000 μg/mL	[8,17]
		*C. glabrata* (1)	>2000 μg/mL	[11]
		*C. krusei* (1)	2000 μg/mL	[11]
		*C. orthopsilosis* (1)	>2000 μg/mL	[11]
		*C. parapsilosis* (1)	>2000 μg/mL	[11]
		*C. tropicalis* (1)	>2000 μg/mL	[11]
*M. leucadendra*	Broth microdilution	*C. albicans* (1)	64–256 μg/mL	[20]
*M. quinquenervia*	Broth microdilution	*C. albicans* (2)	1–4 mg/mL	[11]
*M. quinquenervia*		*C. tropicalis* (1)	1 mg/mL	[11]

* Agar diffusion in mm. ** MIC: Minimum inhibitory concentration (expressed as μg/mL or mg/mL or % (*v*/*v*).

#### 3.2.3. Other Biological Activity of Essential Oils of *Melaleuca* spp.

In vitro studies showed that *M. alternifolia* EO has potent antioxidant activity, the ability to reduce and eliminate superoxide anion radicals [14], and the ability to reduce infectivity against Herpes simplex type 1 (HSV-1) and Herpes simplex type 2 (HSV-2) [21,25].

The EO of *M. alternifolia* has been evaluated for its toxicity to different cell lines and its influence on mediators of the inflammatory process. In vitro studies using MCF-7 and MDA-MB-231 cells derived from breast tumors showed that concentrations greater than 100 μg/mL were toxic [8]. In OKF6-TERT2 cells originating from the oral epithelium, 0.25% (*v*/*v*) demonstrated both a cytotoxic effect and the ability to inhibit the expression of the cytokine IL-8 [24]. In in vivo models using mice with pneumonia induced by *C. albicans*, there was a reduction in the pro-inflammatory mediators IL-1β and TNF-α, as well as a decrease in the recruitment of leukocytes and neutrophils, when inhalable nanoemulsions containing EO of *M. alternifolia* were administered [31]. The *M. leucadendra* EO showed acute toxicity to *Aedes aegypti* and *Cx. quinquefasciatus* larvae, showing repellent potential [20].

### 3.3. Citrus spp.

The genus *Citrus* originates from Southeast Asia and includes about 40 species. It is one of the most important genera of the Rutaceae family and is cultivated in several countries with warm climates [32,33]. Some factors contribute to the known and extensive biological activity of the species of *Citrus*, such as the part of the plant used, plant growth conditions, and the developmental stage at the time of extraction in the case of the fruit, among others [33,34,35,36]. EO can be extracted from the fruit, leaf, and peel, and is used in the composition of fragrances, in cooking, and in the pharmaceutical and cosmetic industries [32,34].

#### 3.3.1. Chemical Composition of Essential Oils from Species of *Citrus* spp.

The main constituents of the EO of *Citrus* spp. described in most publications are limonene, β and α-pinene isomers, and linalool (Supplementary Materials) [8,11,21,34,36,37,38].

The main constituent, limonene, is present in a greater proportion, reaching concentrations of 75.43–90% in the EO of *C. grandis, C. reticulata, C. sinensis, C. paradisi*, and *C. hystrix* [8,21,37]. Intermediate concentrations, however, ranging from 51.09–51.46%, were found for the EO of *C. aurantifolia* [8] and *C. latifolia,* respectively [37]. Other species, such as *C. reticulata* var. Blanco and *C. bergamia*, had concentrations of 34.6% and 37.5%, respectively [8,16].

Other species had different major constituents with varying concentrations. *C. limonum* was the species with the greatest variation in limonene concentration, ranging from 22.4–63.27% [8,11,34]. However, citral was reported to be the main component, reaching a proportion of 53.85% among the constituents, while the proportion of limonene was 5.29% [38]. *C. grandis* had citronellol as the major constituent in the EO extracted from the leaves, which ranged from 30.87–34.54% [36], while the proportion of borneol in the bark was 42.24% [21]. In *C. aurantium*, the major constituents were linalyl acetate and linalool, at levels of 51.5% and 25.4%, respectively [8,39].

#### 3.3.2. Anti-*Candida* Activity of *Citrus* spp. Essential Oil

In many industrial processes involving species of *Citrus*, the peel is not considered, even though it represents about 50% of the fruit [33,36]; however, it is from the peel that EO can be extracted. *Citrus* EO is a potent antimicrobial agent against microorganisms that have considerable importance for human health, such as Gram-negative and Gram-positive bacteria [21], and yeasts such as *Candida* spp. [8,11,28,34].

EOs from different *Citrus* species have been evaluated in vitro against *Candida* species [4,11,21,34,36,37,38,39]. These EOs have a wide spectrum of action against *C. albicans*, in which the in vitro inhibitory activity is variable. The EO of *C. sinensis* and *C. latifolia* showed low action, forming inhibitory halos of 5.51 and 9.46 mm, respectively, when assayed by well diffusion in agar [37]. Still, other findings for *C. sinensis* described an MIC of 625 μg/mL [8] and values greater than 2000 μg/mL [11]. The EO for *C. aurantium* showed an MIC equal to those reported for *C. sinensis,* with an MIC ranging from 0.15–0.31% (*v*/*v*) [8,11,39]. The EO action of *C. hystix* and *C. grandis* was inhibitory against *C. albicans* [21,36]. MICs ranging from 1000–4000 μg/mL and 4000 μg/mL, respectively, for *C. hystix* and *C. grandis* EOs [21], while values from 0.116–0.121% (*v*/*v*) were found for *C. grandis* [36] (Table 2).

EOs from other *Citrus* species have also shown in vitro inhibitory activity against *C. albicans* isolates. MIC variations found according to the studies ranged from 0.0097–3.0% (*v*/*v*) by cylinder-plate diffusion [4,34], and concentrations lower than 0.043–31.325 mg/mL by microdilution in broth [38]. The EO of *C. limon* presented an MIC equal to 500 μg/mL [11] and 625 μg/mL [8]. A similar MIC (625 μg/mL) was observed for the EO of *C. bergamia* and *C. aurantifolia* [8]. The MIC of the EO of *C. reticulata* showed the widest range of variation, from 300 μg/mL to greater than 2000 μg/mL [8,11,21] (Table 2). Meanwhile, MICs of 2000 μg/mL have been related to the EO of *C. nobilis* [11], while there was variation in the MIC for the EO of *C. paradisi* [23] from 0.125–0.25% (*v*/*v*) and an MIC of 313 μg/mL [8].

*C. glabrata* is also susceptible in vitro to *Citrus* EO. For *C. paradisi* EO, the variation in MIC was from 0.0024–1% (*v*/*v*) [4,28]. For *C. limonum,* the range of MICs was lower than 0.043–5.33 mg/mL [38], also demonstrating the concentration-dependent inhibitory activity of *C. limonum* through the cylinder-plate diffusion method against *C. glabrata* (halo formation ranging from 44.6–45 mm) [34]. The EOs of *C. sinensis* and *C. latifolia* showed a halo of 5.78 and 8.52 mm, respectively [37]. MIC ranging from 250 μg/mL to greater than 2000 μg/mL have been reported for EO of *C. limon, C. reticulata, C. nobilis, C. aurantium*, and *C. sinensis* [11] (Table 2).

The EOs of *C. sinensis* and *C. latifolia* inhibited 50% of the growth of *Candida* species, including *C. lusitaniae* (2.00 and 8.06 mm, respectively) and *C. guilliermondii* (only *C. latifolia* was active, 8.94 mm) [37] (Table 2). *C. parapsilosis* and *C. orthopsilosis* were not inhibited in vitro at concentrations of up to 2000 μg/mL with *C. nobilis* and *C. reticulata* EOs, as shown in a previous study [11] (Table 2).

*C. krusei* was inhibited with an MIC between 0.0024 and 0.0019 (% *v*/*v*) when tested in vitro with *C. limonum* EO [4]. However, the EOs of *C. limon, C. sinensis, C. reticulata, C. aurantium*, and *C. nobilis* have shown inhibition ranging from 250 μg/mL to values greater than 2000 μg/mL for *C. krusei* [11] (Table 2).

The EO of *C. limon* presented an MIC of 500 μL/mL for *C. tropicalis* [40], while there were inhibition halos reported between 15.3 and 16.3 mm when they tested *C. limonum* by cylinder-plate diffusion [34]; in contrast, there were inhibition halos ranging from 4.44 to 10.87 mm when using EO of *C. sinensis* and *C. latifolia* by agar diffusion [37] (Table 2). EOs of *C. reticulata, C. aurantium, C. nobilis*, *C. sinensis, C. hystix*, and *C. grandis* have shown an MIC ranging from 1,000 μg/mL to values greater than 4,000 μg/mL by microdilution in broth [11,21].

*Citrus* EOs have also been evaluated for their ability to inhibit and eradicate preformed biofilms. The EO of *C. limon* eradicated 70% or more of the *C. tropicalis* biofilm at concentrations starting from 0.125 x MIC (MIC equal to 500 μL/mL) [40]. Other reports show that 125 μg/mL and 250 μg/mL of the EO of *C. limon*, respectively, were able to inhibit and eradicate the biofilm of *C. krusei* [11].

The EO of *C. limonum* showed the best MIC range for *C. krusei,* from 0.0024–0.0097% (*v*/*v*), for *C. glabrata* from 0.0024–0.1565% (*v*/*v*), and for *C. albicans* from 0.0097–0.312% (*v*/*v*); according to the authors of [4], all isolates were resistant to fluconazole, while there was an MIC of 0.005 and 0.312% (*v*/*v*), respectively, for *C. glabrata* and *C. albicans* [38].

The mechanisms by which the different EOs show inhibitory activity on *Candida* spp. are complex and depend on the chemical constitution and concentration of the major constituents, but usually involve damage to the cell membrane, leading to changes in permeability; however, other cellular activities, such as the disruption of proton pumps, the coagulation of cell contents, leakage of intracellular contents, and consequent apoptosis, necrosis and cell death, have also been reported [38].

**Table 2:** In vitro antifungal activities of *Citrus* spp. essential oils tested against *Candida* species according to different methods.

*Citrus* Species	Method of Antifungal Susceptibility	Species of *Candida* (Number of Strains Tested)	Agar Diffusion * or MIC **	Reference
*C. aurantifolia*	Broth microdilution	*C. albicans* (1)	625 μL/mL	[8]
*C. aurantium*		*C. albicans* (1)	625 μL/mL	
*C. aurantium*	Broth microdilution	*C. albicans* (1)	>2000 μg/mL	[11]
		*C. glabrata* (1)	>2000 μg/mL	
		*C. krusei* (1)	>2000 μg/mL	
		*C. orthopsilosis* (1)	>2000 μg/mL	
		*C. parapsilosis* (1)	>2000 μg/mL	
		*C. tropicalis* (1)	>2000 μg/mL	
*C. aurantium*	Disk diffusion	*C. albicans* (2)	19–25.3 mm	[39]
	Broth microdilution	*C. albicans* (2)	0.15–0.31% (*v*/*v*)	
*C. bergamia*	Broth microdilution	*C. albicans* (1)	625 μL/mL	[8]
*C. grandis*	Broth microdilution	*C. albicans* (1)	4 mg/mL	[21]
		*C. tropicalis* (1)	4 mg/mL	
*C. grandis*	Broth microdilution	*C. albicans* (1)	0.116–0121% (*v*/*v*)	[36]
*C. hystix*	Broth microdilution	*C. albicans* (1)	1–4 mg/mL	[21]
		*C. tropicalis* (1)	2 mg/mL	
*C. latifolia*	Disk diffusion	*C. albicans* (1)	9.46 mm	[37]
		*C. glabrata* (1)	8.52 mm	
		*C. guilliermondii* (1)	8.94 mm	
		*C. lusitaniae* (1)	8.06 mm	
		*C. tropicalis* (1)	10.87 mm	
*C. limon*	Broth microdilution	*C. albicans* (1)	625 μL/mL	[8]
*C. limon*	Broth microdilution	*C. albicans* (1)	500 μg/mL	[11]
		*C. glabrata* (1)	250 μg/mL	
		*C. krusei* (1)	500 μg/mL	
		*C. orthopsilosis* (1)	500 μg/mL	
		*C. parapsilosis* (1)	500 μg/mL	
		*C. tropicalis* (1)	250 μg/mL	
*C. limonum*	Broth microdilution	*C. albicans* (20)	0.0097–0.312% (*v*/*v*)	[4]
		*C. glabrata* (14)	0.0024–0.1565 (*v*/*v*)	
		*C. krusei* (10)	0.0024–0.0097% (*v*/*v*)	
*C. limonum*	Broth microdilution	*C. albicans* (183)	<0.043 to >21.325 mg/mL	[38]
		*C. glabrata* (76)	<0.044 to 5.331 mg/mL	
*C. limonum*	Cylinder-plate diffusion	*C. albicans* (1)	0 mm	[34]
		*C. glabrata* (1)	44.8–45 mm	
		*C. tropicalis* (1)	0 mm	
*C. limonum*	Cylinder-plate diffusion	*C. albicans* (1)	44.8–45 mm	[34]
		*C. glabrata* (1)	0 mm	
		*C. tropicalis* (1)	15.3–16.3 mm	
	Cylinder-plate diffusion	*C. albicans* (1)	23–45.0 mm	[34]
		*C. glabrata* (1)	44.6–44.8 mm	
		*C. tropicalis* (1)	0 mm	
	Cylinder-plate diffusion	*C. albicans* (1)	0 mm	[34]
		*C. glabrata* (1)	0 mm	
		*C. tropicalis* (1)	0 mm	
	Cylinder-plate diffusion	*C. albicans* (1)	0 mm	[34]
		*C. glabrata* (1)	0 mm	
		*C. tropicalis* (1)	0 mm	
C. nobilis	Broth microdilution	*C. albicans* (1)	2000 μg/mL	[11]
		*C. glabrata* (1)	2000 μg/mL	
		*C. krusei* (1)	>2000 μg/mL	
		*C. orthopsilosis* (1)	>2000 μg/mL	
		*C. parapsilosis* (1)	>2000 μg/mL	
		*C. tropicalis* (1)	>2000 μg/mL	
*C. paradisi*	Broth microdilution	*C. albicans* (1)	313 μL/mL	[8]
*C. paradisi*	Broth microdilution	*C. albicans* (30)	0.0039–1% (*v*/*v*)	[23]
*C. paradisi*	Broth microdilution	*C. glabrata* (30)	0.007–1% (*v*/*v*)	[28]
*C. reticulata*	Broth microdilution	*C. albicans* (1)	625 μL/mL	[8]
*C. reticulata*	Broth microdilution	*C. albicans* (1)	2000 μg/mL	[11]
		*C. krusei* (1)	250 μg/mL	
		*C. glabrata* (1)	1000 μg/mL	
		*C. parapsilosis* (1)	1000 μg/mL	
		*C. orthopsilosis* (1)	250 μg/mL	
		*C. tropicalis* (1)	1,000 μg/mL	
*C. reticulata* var. Blanco	Broth microdilution	*C. albicans* (1)	1.00 to > 2000 μg/mL	[11]
		*C. krusei* (1)	500 to >2000 μg/mL	
		*C. glabrata* (1)	1000–2000 μg/mL	
		*C. parapsilosis* (1)	1000–2000 μg/mL	
		*C. orthopsilosis* (1)	1000–2000 μg/mL	
		*C. tropicalis* (1)	2.00 to > 2000 μg/mL	
*C. reticulata* Blanco var. *cravo*	Broth microdilution	*C. albicans* (1)	2.00 to > 2000 μg/mL	[11]
		*C. krusei* (1)	>2000 μg/mL	
		*C. glabrata* (1)	>2000 μg/mL	
		*C. parapsilosis* (1)	>2000 μg/mL	
		*C. orthopsilosis* (1)	>2000 μg/mL	
		*C. tropicalis* (1)	2.00 to >2000 μg/mL	
*C. reticulata* var. Blanco	Broth microdilution	*C. albicans* (1)	0.3–4 mg/mL	[21]
		*C. tropicalis* (1)	2 mg/mL	
*C. sinensis*	Disk diffusion	*C. albicans* (1)	5.51 mm	[37]
		*C. glabrata* (1)	5.78	
		*C. lusitaniae* (1)	2.00	
		*C. tropicalis* (1)	4.44 mm	
*C. sinensis*	Broth microdilution	*C. albicans* (1)	>2000 μg/mL	[11]
		*C. krusei* (1)	>2000 μg/mL	
		*C. glabrata* (1)	>2000 μg/mL	
		*C. parapsilosis* (1)	>2000 μg/mL	
		*C. orthopsilosis* (1)	>2000 μg/mL	
		*C. tropicalis* (1)	>2000 μg/mL	

* Agar diffusion in mm. ** MIC: Minimum inhibitory concentration (expressed as μg/mL or mg/mL or % (*v*/*v*).

#### 3.3.3. Other Biological Activity of Essential Oils of *Citrus* spp.

*Citrus* EOs have other biological activities, as shown by in vitro studies, such as antioxidant, anti-inflammatory, and anti-pigmentation. These activities are present in the EO of *C. grandis*, which make it an option for the development of dermatological products, in which in vitro studies have shown effectiveness at concentrations lower than 0.05% (*v*/*v*) [33,36].

By computational modeling (in silico) [39], the potential use of *C. aurantium* EO as an antimicrobial agent has been suggested in in vivo models of infection, such as *Caenorhabditis elegans* [11]. On the other hand, the EO of *C. limon* showed toxicity to larvae of *C. elegans*, even at the same concentration as that which was effective in vitro against *C. tropicalis* [11].

The in vitro toxicity of *Citrus* EOs varied according to the different cells assayed, such as human breast cancer cell lines and human oral epithelium [8,28,37]. Most EOs tested showed toxicity above 50 μg/mL in MDA-MB-231 and MCF-7 breast cancer cells [8], and 21.8 μg/mL for *C. latifolia* in human oral epithelial cells [37].

### 3.4. Cupressus spp.

The genus *Cupressus* is native to the northern hemisphere and includes more than ten species and variants [41]. Plants of this genus are cultivated in a temperate climate, which is attractive for ornamental purposes and wood extraction, and are distributed in commercial plantations all over the world [42]. A wide spectrum of biological activities has been attributed to substances present in its aerial parts, including in the EO [43]. In folk medicine, cypress EO acts as an antispasmodic for coughing, as a diuretic, and in the improvement of affections of the venous and renal circulation, in addition to acting on inflammatory processes and against infectious microorganisms [10,41,42,44].

#### 3.4.1. Chemical Composition of Essential Oils from Species of *Cupressus* spp.

*Cupressus* EO is usually extracted from aerial parts and leaves, and the chemical composition varies according to the species and study. The main component is α-pinene, found in *C. arizonica* (26.53–29.76%) [42], *C. lusitanica* (13.8–35.7%) [44], *C. macrocarpa* (63.2%) [21], and in *C. sempervirens* (4.6–49.7%) [8,10,11]. Other constituents, such as δ-3-carene, terpinen-4-ol, limonene, sabinene, umbellulone, α-thujene, and cedrol, appear in smaller proportions and vary according to species [8,21] (Supplementary Materials).

As in all essential oils in general, the factors that influence the different proportions of constituents include the location/region of cultivation, the part of the plant collected, the period of plant development in the EO extraction, and varieties of the species [6,8,10,11,42,44].

#### 3.4.2. Anti-*Candida* Activity of *Cupressus* spp. Essential Oil

*Cupressus* EOs have an anti-*Candida* inhibitory effect demonstrated by in vitro studies, which vary according to the yeast species but also according to the plant species [6,42], (Table 3). The evaluation of the inhibitory effect of EOs against *C. albicans*, according to the methodology used, showed that the species *C. arizonica*, *C. sempervirens, C. lusitanica*, and *C. macrocarpa* have inhibitory activities in some way by different concentrations of EO. Evaluating the same *Candida* species by microdilution, the inhibitory activity of EO of *C. arizonica* was expressed by an MIC of 0.05 μL/mL [42] and was also expressed at 0.42 ± 0.027 μL/mL for the EO of *C. sempervirens* [10]. Other studies found an MIC of 625 μg/mL for *C. sempervirens* [8] and 2000 μg/mL for *C. macrocarpa* [21]. For the EO of *C. lusitanica* against *C. albicans*, both MIC and CFM were equal to 0.16% (*v*/*v*) [6]. By using the agar diffusion disk technique, a 13.0 mm halo was produced when using a 10 μL/100% (*v*/*v*) *C. lusitanica* EO disk [6]; for this same species, there were inhibition halos of 7.5 to 8.5 mm when 1.5 μL of EO/disks of *C. lusitanica* were placed [44] (Table 3).

For *C. glabrata*, the EO of *C. arizonica* presented an MIC ranging between 0.01 and 0.05 μL/mL [42], the EO of *C. lusitanica* presented an MIC of 1.25% (*v*/*v*) [6], and the EO of *C. sempervirens* presented an MIC of 31.25μg/mL [11].

*C. krusei* was tested with EOs of *C. sempervirens* and *C. lusitanica*. The MIC for *C. sempervirens* was 62.5 μg/mL [11], and for *C. lusitanica* it was 1.25% (*v*/*v*); the halos were 10 mm when using disks containing 10 µL of the EO [6].

*Cupressus* species showed variable results for *C. parapsilosis*. This *Candida* species was inhibited by an MIC ranging from 0.01–0.05 μL/mL when assayed with the EO of *C. arizonica* [42]. Using the disk diffusion technique, the EO of *C. lusitanica* obtained an MIC of 1.25% (*v*/*v*) and halos of 7.0 mm [6]. Assessing *C. parapsilosis* and *C. orthopsilosis*, the MICs were found to be 62.5 μg/mL and 31.25 μg/mL, respectively [11] (Table 3).

For *C. tropicalis*, *C. arizonica*, and varieties, the MIC ranged from 0.001–0.01 μL/mL [42], for *C. sempervirens* it was 250 μg/mL [11], and for *C. macrocarpa* it was 2000 μg/mL [21]. The EO of *C. lusitanica* inhibited *C. arizonica* at a concentration of 1.25% (*v*/*v*) and presented halos of 14.0 mm, when they used disks containing 10 μL of the EO [6].

For *C. lusitaniae*, the EO of *C. lusitanica* presented an MIC of 0.62 μg/mL and halos of 13.0 mm in disks containing 10 μL of the EO [6]. Other *Candida* species, such as *C. bracarensis* and *C. dubliniensis*, were inhibited by concentrations ranging from 0.01–0.05 μL/mL when the EO of *C. arizonica* and varieties were evaluated [42] (Table 3).

#### 3.4.3. Biological Activity of Essential Oils of *Cupressus* spp.

In vitro studies have reported different activities of *Cupressus* EOs, as reported for the antioxidant and anti-inflammatory activity of *C. lusitanica* [44]. An in vivo study using a murine model (Swiss mice and albino Wistar rats) showed a lethal dose of 6.33 g/kg [6].

The toxicity evaluation of *C. sempervirens* EO using human breast cancer cell lines (MCF-7 and MDA-MB-231) showed a 50% inhibition of cell viability at concentrations of 34.5 μg/mL and 65.2 μg/mL, respectively, for MCF-7 and MDA-MB-231 lineages [8]. In another study, 60% of *C. elegans* larvae infected with *C. glabrata* survived after four days of exposure to *C. sempervirens* EO at a concentration of 62.5 μg/mL [11].

### 3.5. Litsea spp.

About 400 species of *Litsea* have been described around the world; *L. cubeba* is one of the most well-studied, due to its antimicrobial, anti-inflammatory, and immunomodulatory activities [45], but also for its commercial value, with the countries India, Taiwan, Japan, and China being the largest producers and exporters of *L. cubeba* EO worldwide [46]. In general, EOs of *Litsea* have a fresh, sweet, citrus aroma, are insoluble in water, and are widely used in traditional medicine [45,46].

#### 3.5.1. Chemical Composition of *Litsea* Species

The composition of *Litsea* EOs varies, as for all EOs from different plants, according to species of the plant, the part of the plant from which they are extracted, and the region and country of origin. Two species of *Litsea* included in this study had their chemical composition detailed: *L. cubeba* and *L. viridis* (Supplementary Materials).

The composition of the EO of *L. viridis*, extracted from the leaves of the plant collected in Vietnam, includes bicyclogermacrene (25.5%), decanal (14.4%), α-pinene (11.1%), β-pinene (8.3%), and aromadendrene (3%) as the most frequent compounds [47].

In Brazil, limonene (37%), neral (31.4%), and citral (12%) were the most frequent compounds in the EO extracted from the fruits of *L. cubeba* [11]. However, other review studies on the EO of *L. cubeba* extracted from plants cultivated in other countries revealed a diverse chemical composition, with a predominance of 1,8-cineole, sabinene, and α-pinene in the leaves [9], and citral, citronellol, citronellal, geranial, limonene, linalool, neral, α-pinene, and β-pinene in the EO extracted from fruits [46] (Table 3).

#### 3.5.2. Anti-*Candida* Activity of *Litsea* spp. Essential Oil

The in vitro inhibitory effect of *L. viridis* EO showed an MIC of 128 μg/mL for *C. albicans* [46], and that of *L. cubeba* showed an MIC equal to 500 μg/mL for *C. albicans* [11] (Table 3).

**Table 3:** In vitro antifungal activities of *Cupressus* spp. and *Litsea* spp. essential oils tested against *Candida* species according to different methods.

*Melaleuca* Species	Method of Antifungal Susceptibility	Species of *Candida* (Number of Strains Tested)	Agar Diffusion * or MIC **	Reference
*C. lusitanica*	Disk diffusion	*C. albicans* (2)	6–13 mm	[6,44]
		*C. glabrata* (1)	6 mm	[6]
		*C. krusei* (1)	6–10 mm	[6]
		*C. lusitaniae* (1)	6–13 mm	[6]
		*C. parapsilosis* (1)	6–7 mm	[6]
		*C. tropicalis* (1)	6–14 mm	[6]
	Macrowell dilution	*C. albicans* (1)	0.16% (*v*/*v*)	[6]
		*C. glabrata* (1)	1.25% (*v*/*v*)	[6]
		*C. krusei* (1)	1.25% (*v*/*v*)	[6]
		*C. lusitaniae* (1)	0.62% (*v*/*v*)	[6]
		*C. parapsilosis* (1)	1.25% (*v*/*v*)	[6]
		*C. tropicalis* (1)	1.25% (*v*/*v*)	[6]
*C. arizonica* var. *glabra*	Broth mcrodilution	*C. albicans* (1)	0.05 μL/mL	[42]
		*C. dublinenensis* (1)	0.01 μL/mL	[42]
		*C. glabrata* (1)	0.05 μL/mL	[42]
		*C. parapsilosis* (1)	0.05 μL/mL	[42]
		*C. tropicalis* (1)	0.001 μL/mL	[42]
*C. arizonica* var. *arizonica*	Broth mcrodilution	*C. albicans* (1)	0.05 μL/mL	[42]
		*C. dublinenensis* (1)	0.01 μL/mL	[42]
		*C. glabrata* (1)	0.01 μL/mL	[42]
		*C. parapsilosis* (1)	0.01 μL/mL	[42]
		*C. tropicalis* (1)	0.01 μL/mL	[42]
*C. sempervirens*	Broth microdilution	*C. albicans* (1)	0.42 ± 0.027 μL/mL	[10]
		*C. glabrata* (1)	<64 μL/mL	[10]
		*C. krusei* (1)	<64 μL/mL	[10]
		*C. parapsilosis* (1)	0.757 ± 0.067 μL/mL	[10]
*C. sempervirens*	Broth microdilution	*C. albicans* (2)	250–625 μg/mL	[8,11]
		*C. glabrata* (1)	31.25 μg/mL	[11]
		*C. krusei* (1)	62.5 μg/mL	[11]
		*C. orthopsilosis* (1)	31.25 μg/mL	[11]
		*C. parapsilosis* (1)	62.5 μg/mL	[11]
		*C. tropicalis* (1)	250 μg/mL	[11]
*C. macrocarpa*	Broth microdilution	*C. albicans* (2)	1–2 mg/mL	[21]
		*C. tropicalis* (1)	2 mg/mL	[21]
*L. viridis*	Broth microdilution	*C. albicans* (1)	128 μg/mL	[47]
*L. cubeba*	Broth microdilution	*C. albicans* (1)	500 μg/mL	[11]
		*C. krusei* (1)	62.5 μg/mL	[11]
		*C. glabrata* (1)	250 μg/mL	[11]
		*C. orthopsilosis* (1)	250 μg/mL	[11]
		*C. parapsilosis* (1)	500 μg/mL	[11]
		*C. tropicalis* (1)	1000 μg/mL	[11]

* Agar diffusion in mm. ** MIC: Minimum inhibitory concentration (expressed as μg/mL or mg/mL or % (*v*/*v*).

The in vitro inhibitory effect of *L. cubeba* EO was evaluated in biofilm formation and performed biofilm eradication for *C. albicans* and non-*albicans Candida* species such as *C. glabrata*, *C. orthopsilosis*, and *C. tropicalis* [11]. Thus, they found that EOs at concentrations of 2000 and 1000 μg/mL were able to, respectively, inhibit biofilm formation and eliminate biofilms for most of the species. For *C. parapsilosis*, both MBIC and MBEC were 1000 μg/mL, whereas the MBIC and MBEC for *C. krusei* were 250 and 1000 μg/mL, respectively [11].

#### 3.5.3. Other Biological Activity of Essential Oils of *Litsea* spp.

The toxicity of *Litsea cubeba* EO was evaluated in an in vivo *C. elegans* model and showed no toxic effects at concentrations up to 125 μg/mL following 24 h of exposure [11].

## 4. Discussion

This systematic review presented an evaluation of the in vitro anti-*Candida* inhibitory effect of essential oils from *Melaleuca, Citrus*, *Litsea*, and *Cupressus*. Several factors interfere with the chemical composition of the EO, including the origin of the plant, as well as the location and growing conditions, seasonal variation, phenotypic variation, and the part of the plant from which the EO was extracted. This variation is even greater when comparing the EO from different species of the same genus. In addition, the predominance of certain chemical constituents in the EO can determine its greater or lesser effectiveness [33,34,35,36,48].

The in vitro determination of antifungal inhibitory effect is performed by different techniques. According to the publications analyzed, there was a predominance of the broth dilution methodology, using techniques whose results were expressed as % (*v*/*v*), μg/mL, and μL/mL. In recent years, the use of more sensitive methodologies in the evaluation of potential antimicrobial agents has shown that techniques based on agar diffusion have been replaced by microdilution in broth [49,50]. For this reason, a comparison of the results between studies was one limitation due to the lack of standardization of the methodologies used. The diversity of methodologies compromises an accurate analysis of the results, often allowing evidence of in vitro antimicrobial activity, without analyzing to any extent the reason why EO from different origins and different studies show variable results. Thus, it is not defined which factors can influence the results of in vitro tests and how much, such as the origin and chemical composition of the oil, the particularity of the tested isolates, technical conditions whose tests were performed, and the solvent used to dilute the EO [6,44].

The antifungal activity of EO of *Melaleuca*, mainly *M. alternifolia*, has been extensively studied for *Candida* species, and there seem to be no major differences in responses for EOs of *Melaleuca* for different isolates, regardless of the EO origin [8,11,15,21,23,24,25,27]. In addition, other species of *Melaleuca* have shown the potential inhibition of *Candida* spp., especially for EOs extracted from leaves and aerial parts. This may expand to the EOs extracted from other parts of the plant, which requires further investigation [20].

On the other hand, *Citrus* presents an extensive variability of EO-producing according to species. This allows the comparison of inhibitory activity against *Candida* spp. and also enables the evaluation of anti-*Candida* activity among EO extracted from different parts of the plant [8,11,21,37].

*Cupressus* EOs were evaluated by different methodologies and showed antifungal effects against many of the *Candida* species. The techniques employed in the in vitro evaluations of EOs can also, in addition to determining the in vitro susceptibility of fungi to antifungal drug candidates, be considered for the research and development of new strategies of use, such as for evaluating the synergism between different natural products and between them and the already known antifungal drugs [7,38,39,51].

The EOs of the two *Litsea* species have been evaluated. They showed in vitro anti-*Candida* activity, including on biofilm (*L. cubeba*), in a study that tested *C. glabrata* and *C. krusei*, species with limited susceptibility or resistance to fluconazole, one of the azole drugs that is most commonly used for the treatment of *Candida* spp. [11,45].

Thus, considering the great diversity of *Litsea* species, it will be of substantial importance to explore the EOs of the other species, grown in different regions throughout the world, in determining the chemical constitution and performing biological studies, including searches for antifungal activities [44,46].

The increasing discoveries in the field of natural products, and the development and improvement of technologies in the pharmaceutical field, which enable the incorporation of drugs into nanoparticles and nanodispersions, can promote the optimization of the activity profile of several drugs. Effective and safe nanodispersion technologies can circumvent the limitations of hydrophobicity, volatility, and other therapeutic adversities attributed to the loss of physical–chemical stability in formulations containing EOs, mainly applied in formulations aimed at the treatment of superficial candidiasis [4,22,27,30].

In recent years, it has become clear that there is still much to be studied regarding EO. Ethnobotany and ethnopharmacology can contribute considerably to this field. The chemical composition of oils, both qualitative and quantitative, is very variable, and the combination of different molecules, from different classes, in a single oil can result in characteristics that act differently in biological systems. Thus, an understanding of the associated antioxidant, anti-inflammatory, antimicrobial properties, in addition to others involving the field of aromatherapy study, may contribute substantially to the treatment of problems that affect humans, such as infections caused by *Candida* species.

## 5. Conclusions

Infections caused by *Candida* spp. mainly involve patients with comorbidities. The increasing number of patients in immunocompromised conditions or with bacterial and viral coinfections or other opportunistic fungi has made treatment with conventional antifungal agents a challenge. Therefore, innovative research is being developed to understand which EO molecules have relevant biological activity for application in the treatment of fungal infections. Technologies that enable the incorporation of EOs in pharmaceutical formulations can improve the active release profile. Thus, this is a field with growing potential for future studies. In conclusion, this study showed in vitro evidence for the use of *Melaleuca, Cupressus, Citrus*, and *Litsea* EOs for the treatment of infections caused by different *Candida* species.

## Supplementary Materials

The following are available online at https://www.mdpi.com/article/10.3390/pharmaceutics13101700/s1: Table S1: Anti-*Candida* Activity of *Melaleuca* spp. essential oils and their main components, Table S2: Anti-*Candida* Activity of *Citrus* spp. essential oils and their main components, Table S3: Anti-*Candida* Activity of *Cupressus* spp. essential oils and their main components, Table S4: Anti-*Candida* Activity of *Litsea* spp. essential oils and their main components.
