# Supplementary material for: Essential Oils of Melaleuca, Citrus, Cupressus, and Litsea for the Management of Infections Caused by Candida Species: A Systematic Review
Source: Pharmaceutics. 2021 Oct 15;13(10):1700. doi: 10.3390/pharmaceutics13101700 (PMC8540016; doi:10.3390/pharmaceutics13101700)
Supplement: Supplementary file 1 [file pharmaceutics-13-01700-s001.zip › pharmaceutics-1333157-supplementary.pdf]

pharmaceutics

MDPI

Supplementary Materials: Essential Oils of *Melaleuca, Citrus*, *Cupressus*, and *Litsea* for the Management of Infections Caused by *Candida* Species: A Systematic Review

Rafael Alves da Silva, Flávia Maria Pinto Monteiro Antonieti, Denise Von Dolinger de Brito Röder and Reginaldo dos Santos Pedroso

Table S1. Anti-*Candida* Activity of *Melaleuca* spp. essential oils and their main components.

Melaleuca Species	Part of the Plant	Antifungal Activity					Major Components (>1%)	Reference
		Method of Antifungal Susceptibility	Species of *Candida*	Agar Diffusion or MIC *	MFC **	Comparison Drug Results		
M. alternifolia	Leaves	Disk difusion	*C. albicans* (RCMB 05035)	20.3 ± 0.44 mm	-	Amphotericin B (5 µg/mL/disk): 21.9 ± 0.12 mm	Terpinen-4-ol (44.41%), γ-terpinene (21.89%), α-terpinene (6.84%), α-terpineol (6.46%), α-pinene (2.87%), limonene (2.86%), aromadendrene (2.86%), o-cymene (o-cymol) (2.81%), Terpinolene (2.45%), eucalyptol (1,8-cineole) (1.31%).	[1]
M. alternifolia *M. alternifolia*	Leaves	Disk difusion	*C. albicans* (ATCC 90028)	(30 mg/10 µL/ disk): 19.33 ± 0.57 mm	-	Amphotericin B (10 µg/disk): 11 mm	Terpinen-4-ol (40.44%), γ-terpinene (19.54%), α-terpinene (7.69%), 1,8-cineole (5.20%), *þ* -cymene (4.74%), α-terpineol (3.31%), α-terpinolene (3.09%), α-pinene (2.67%).	[2]
			C. albicans (ATCC 2091)	(30 mg/10 µL/ disk): 21 ±1 mm	-	Amphotericin B (10 µg/disk): 14.66 ± 0.57 mm		
			C. albicans	(30 mg/10 µL/ disk): 12–25 mm	-	Amphotericin B (10 µg/disk): 9.66–11.66 mm		
			C. atlantica (CECT 1435)	(30 mg/10 µL/ disk): 21 ± 1 mm	-	Amphotericin B (10 µg/disk): 11 ± 0 mm		
			C. dublinenensis (CECT 11455)	(30 mg/10 µL/ disk): 15 ± 0 mm	-	Amphotericin B (10 µg/disk): 11.33±0.57 mm		
			C. famata (CECT 11957)	(30 mg/10 µL/	-	Amphotericin B		

				disk): 20.66 ± 0.57 mm		(10 µg/disk): 12.33±0.57 mm
			C. glabrata (ATCC 90030)	(30 mg/10 µL/ disk): 14.33 ± 0.57 mm	-	Amphotericin B (10 µg/disk): 14.33 ± 0.57 mm
			C. glabrata	(30 mg/10 µL/ disk): 11.66–12 mm	-	Amphotericin B (10 µg/disk): 10.66 ± 10.33 mm
			C. intermedia (CECT 11869)	(30 mg/10 µL/ disk): 20 ± 0 mm	-	Amphotericin B (10 µg/disk): 12 ± 0 mm
			C. kefyr (CECT 1017)	(30 mg/10 µL/ disk): 19.33 ± 1.15 mm	-	Amphotericin B (10 µg/disk): 10.66 ± 1.15 mm
			C. kefyr	(30 mg/10 µL/ disk): 25.3 ± 0.57 mm	-	Amphotericin B (10 µg/disk): 9.66 ± 0.57 mm
			C lusitaniae (CECT 11455)	(30 mg/10 µL/ disk): 15.33 ± 0.57 mm	-	Amphotericin B (10 µg/disk): 12.33 ± 1.15 mm
			C. maritima (CECT 1435)	(30 mg/10 µL/ disk): 24.66 ± 0.57 mm	-	Amphotericin B (10 µg/disk): 12.66 ± 1.15 mm
			C. parapsilosis	(30 mg/10 µL/ disk): 14.66 ± 0.57 mm	-	Amphotericin B (10 µg/disk): 11 ± 1 mm
			C. sake (CECT 1044)	(30 mg/ 10 µL/disk): 16.33±0.57 mm	-	Amphotericin B (10 µg/disk): 12 ± 1 mm
M. alternifolia	Leaves	Broth microdilution	*C. albicans* (ATCC 90028)	0.312 mg/mL	>10 mg/mL	Amphotericin B (MIC): 0.781 mg/mL
			C. albicans (ATCC 2091)	0.0097 mg/mL	>10 mg/mL	Amphotericin B (MIC): 0.781 mg/mL
			C. albicans	0.0097–5 mg/mL	5 to > 10 mg/mL	Amphotericin B (MIC): 0.04–1.562 mg/mL
			C. atlantica CECT 1435	0.0097 mg/mL	10 mg/mL	Amphotericin B (MIC): 0.0097 mg/mL
			C. dublinensis	0.0195 mg/mL	5 mg/mL	Amphotericin B (MIC):

						0.0097 mg/mL		
			C. famata CECT 11957	0.0097 mg/mL	>10 mg/mL	Amphotericin B (MIC): 0.0097 mg/mL		
			C. glabrata (ATCC 90030)	0.625 mg/mL	10 mg/mL	Amphotericin B (MIC): 1.562 mg/mL		
			C. glabrata	0.0097 – 0.0195 mg/mL	2.5–10 mg/mL	Amphotericin B (MIC): 0.195–0.39 mg/mL		
			C. intermedia CECT 11869	0.0097 mg/mL	> 10 mg/mL	Amphotericin B (MIC): 0.0097 mg/mL		
			C. *kefyr* (CECT 1017)	0.0097 mg/mL	5 mg/mL	Amphotericin B (MIC): 0.39 mg/mL		
			C. kefyr	0.0097 mg/mL	10 mg/mL	Amphotericin B (MIC): 1.562 mg/mL		
			C. lusitaniae	0.0097 mg/mL	>10 mg/mL	Amphotericin B (MIC): 0.0097 mg/mL		
			C. maritima CECT 1435	0.0097 mg/mL	10 mg/mL	Amphotericin B (MIC): 0.0097 mg/mL		
			C. parapsilosis	5 mg/mL	>10 mg/mL	Amphotericin B (MIC): 0.195 mg/mL		
			C. sake CECT 1044	0.0097 mg/mL	10 mg/mL	Amphotericin B (MIC): 0.0097 mg/mL		
M. alternifolia	Leaves	Broth microdilution	*C. albicans* (ATCC 10231)	0.125% (*v/v*)	0.25% (*v/v*)	Fluconazole (MIC): 256 µg/mL; (MFC): 256 µg/mL	α-Pinene (2.5%), sabinene (0.1%), α-terpinene (8.1%), limonene (1%), þ -cymene (4.4%), 1,8-cineole (2.8%), γ-terpinene (19.6%), terpinolene (3.2%), terpinen-4-ol (41%), α-terpineol (3%), aro-madendrene (1.3%).	[3]
			C. albicans	0.19% (*v/v*)	0.37% (*v/v*)	Fluconazole (MIC): 244 µg/mL; (MFC): 254.48 µg/mL		
M. alternifolia	-	Broth microdilution	*C. albicans*	0.25–2.0% (*v/v*)	-	-	Terpinen-4-ol (41.9%), γ-terpinene (17.8%), α-terpinene (8%), þ -cymene (4.6%), 1,8-cineole (4.4%), α-terpineol (3.8%), α-terpinolene (3%), α-pinene (2.4%), limo-nene (1.8%), ledene (1.2%).	[4]
			C. albicans – Fluconazole Resistant	0.5% (*v/v*)	-	-		
			C. albicans – Fluconazole, voriconzol Resistant	0.12–1.0% (*v/v*)	-	-		
			C. albicans - Fluconazol, Nystatin and Voriconazol Resistant	1% (*v/v*)	-	-		
			C. boidinii - Fluconazole Resistent	0.12% (*v/v*)	-	-		

			C. boidinii	0.12–0.25% (*v*/*v*)	-	-		
			C. colliculosa	0.25% (*v*/*v*)	-	-		
			C. famata	0.25–0.5% (*v*/*v*)	-	-		
			C. glabrata - Fluconazole, voriconazol Resistant	0.25% (*v*/*v*)	-	-		
			C. krusei	0.12–0.25% (*v*/*v*)	-	-		
			C. lusitaniae	0.25–1.0% (*v*/*v*)	-	-		
			C. pelliculosa - Nystatin Resistant	0.5% (*v*/*v*)	-	-		
			C. rugosa – Fluconazole Resistant	0.12% (*v*/*v*)	-	-		
			C. tropicalis - Fluconazol, Nystatin and Voriconazol Resistent	8% (*v*/*v*)	-	-		
M. alternifolia	-	Broth microdilution	*C. albicans* (ATCC 18804)	625 μg/mL	-	-	Terpinen-4-ol (47.5%), γ -terpinene (20.2%), α-terpinene (8.6%).	[5]
M. alternifolia	Leaves and twigs	Broth microdilution	*C. albicans* (NYCY 1363)	0.2% (*v*/*v*) / 1.8 (g/L)	-	-	-	[6]
			C. albicans (135 BM2/94)	0.2% (*v*/*v*) /1.8 (g/L)	-	-		
M. alternifolia	-	Broth microdilution	*C. albicans* (ATCC 10231)	0.5% (*v*/*v*)	-	-	Terpinen-4-ol (41.9%), γ-terpinene (17.8%), α-terpinene (8%), *p* -cymene (4.6%), 1,8-cineole (4.4%), α-terpineol (3.8%), α-terpinolene (3%), α-pinene (2.4%), limonene (1.8%), ledene (1.2%).	[7]
M. alternifolia	-	Broth microdilution	*C. albicans* (ATCC 18804)	0.195% (*v*/*v*) (1.95 mg/mL)	-	-	Terpinen-4-ol (42.8%), γ-terpinene (20.4%), *p* -cymene (9.6%), α-terpinene (7.9%), 1,8-cineole (3%), α-terpineol, (2.8%), α-pinene (2.4%).	[8]
M. alternifolia	-	Broth microdilution	*C. albicans* (ATCC 14053)	2–4% (*v*/*v*)	-	Clotrimazole (MIC): >64 μg/mL Fluconazole (MIC): >64 μg/mL Itraconazole (MIC): >64 μg/mL	α-Terpinen (8.35%), 1,8-cineole (3.39%), α-terpineole (2.7%), α-pinene (2.63 %), aromadendrene (1.65%).	[9]

M. alternifolia	-	Broth microdilution	*C. albicans*	0.50–1% (*v*/*v*)	-	-	-	[10]
M. alternifolia	-	Broth microdilution	*C. albicans* (TIMM 3163)	20 mg/mL (20,000 μg/mL)	-	Fluconazole (MIC): 1 μg/mL	Terpinen-4-ol (37.7%), γ-terpinene (21.25%), α-terpinene (10.5%), terpinolene (3.65%), 1–8 cineole (3.65%), α-terpinenol (2.75%), α-pinene (2.65%), *þ* -cymene (2.3%).	[11]
			C. albicans (TIMM 2640) - Fluconazole Resistant	5 mg/mL (5000 μg/mL)	-	Fluconazole (MIC): >64 μg/mL		
M. alternifolia	-	Broth microdilution	*C. albicans* – Fluconazole resistant	0.156–1.25% (*v*/*v*)	0.312–1.25% (*v*/*v*)	-	-	[12]
			C. glabrata – Fluconazole resistant.	0.156–1.25% (*v*/*v*)	0.625–1.25% (*v*/*v*)	-		
			C. krusei– Fluconazole resistant.	0.312–0.625% (*v*/*v*)	0.312–1.25% (*v*/*v*)	-		
M. alternifolia	-	Broth microdilution	*C. glabrata* (ATCC 2950)	0.75–2.5% (*v*/*v*)	-	Amphotericin B (MIC): 0.0018% (*v*/*v*)	Terpinen-4-ol (40–50% (*v*/*v*)); γ-terpinene (15–25% (*v*/*v*)); α-terpinene (5–15% (*v*/*v*)); Limonene + 1,8-cineole (5–10% (*v*/*v*)); α-pinene (0–10% (*v*/*v*)), α-terpinen-4-ol (0–10% (*v*/*v*)); p -cymene (5–<10% (*v*/*v*)); terpinolene (0–<5% (*v*/*v*)).	[13]
M. alternifolia	-	Broth microdilution	*C. glabrata* (ATCC 15126)	-	-	Clotrimazole (MIC): 8 μg/mL Fluconazole (MIC): 8 μg/mL Itraconazole (MIC): 1 μg/mL	-	[14]
M. alternifolia	-	Broth microdilution	*C. glabrata*	4% (*v*/*v*)	-	Clotrimazole (MIC): 0.062–8 μg/mL Fluconazole (MIC): 2–>64 μg/mL Itraconazole (MIC): 0.125–>64 μg/mL		
M. alternifolia	-	Broth microdilution	*C. krusei*	0.5% (*v*/*v*)	0.5% (*v*/*v*)	-	-	[15]
M. alternifolia	-	Broth microdilution	*C. albicans* (SC 5314)	>2000 μg/mL	-	-	-	[16]
			C. glabrata (ATCC 2001)	>2000 μg/mL	-	-		

			*C. krusei (*ATCC 62580)	2000 μg/mL	-	-		
			C. orthopsilosis (ATCC 96141)	>2000 μg/mL	-	-		
			C. parapsilosis (ATCC 22019)	>2000 μg/mL	-	-		
			C. parapsilosis (ATCC 96141)	>2000 μg/mL	-	-		
			C. tropicalis (ATCC 13803)	>2000 μg/mL	-	-		
M. leucadendra	Young Leaf	Broth microdilution	*C. albicans* (ATCC 10231)	-	-	Nystatin (MIC): 8 μg/mL	*α*-Eudesmol (21.2%), guaiol (12.5%), bulnesol (5.3%), Linalool (4.9%), *p* -cymene (3.9%), *γ*-eudesmol (3.9%), (E)-caryophyllene (3.8%), cis-cadin-4-en-7-ol (3.5%), eremoligenol (3.4%), terpinolene (3%), *α*-humulene (2.8%), β-selinene (2.4%), *γ*-terpinene (2.2%), *α*-selinene (2.1%), selina-6-en-4β-ol (2%), selin-11-en-4*α*-ol (1.9%), caryophyllene oxide (1.8%), *p* -cymen-8-ol (1%), δ-selinene (1%), hinesol (1%).	[17]
M. leucadendra	Old Leaf	Broth microdilution	*C. albicans* (ATCC 10231	128 μg/mL	-	Nystatin (MIC): 8 μg/mL	*α*-eudesmol (17.6%), guaiol (10.9%), (E)-caryophyllene (7%), 1,8-cineole (5.2%), linalool (5.1%), *α*-humulene (4.4%), β-selinene (3.7%), *α*-selinene (3.7%), bulnesol (3.6%), eremoligenol (3.4%), cis-cadin-4-en-7-ol (3%), *γ*-eudesmol (2.8%), caryophyllene oxide (2.3%), *α*-terpineol (1.8%), *p* -cymene (1.7%), terpinolene (1.6%), selina-6-en-4β-ol (1.6%), δ-selinene (1.6%), selin-11-en-4*α*-ol (1.5%), *γ*-terpinene (1.3%), *α*-amorphene (1.2%), *γ*-gurjunene (1.1%).	
M. leucadendra	Stem Bark	Broth microdilution	*C. albicans* (ATCC 10231	64 μg/mL	-	Nystatin (MIC): 8 μg/mL	*α*-eudesmol (24.1%), guaiol (11.3%), (E)-caryophyllene (5.5%), eremoligenol (4.9%), β-selinene (4.8%), *α*-selinene (3.6%), *α*-humulene (3.5%), *γ*-eudesmol (3.5%), bulnesol (3.3%), cis-cadin-4-en-7-ol (3.3%), caryophyllene oxide (3.3%), 1,8-cineole	

							(1.8%), selina-6-en-4β-ol (1.7%), α-amorphene (1.5%), humulene epoxide II (1.5%), linalool (1.4%), limonene (1.4%), *p* -cymene (1.3%), selin-11-en-4α-ol (1.3%), hinesol (1.2%), γ-gurjunene (1.1%),	
M. leucadendra	Fruit	Broth microdilution	*C. albicans* (ATCC 10231	256 μg/mL	-	Nystatin (MIC): 8 μg/mL	α-eudesmol (30.7%), guaiol (10.4%), eremoligenol (6.5%), γ-eudesmol (5.3%), bulnesol (4.4%), (E)-caryophyllene (4.3%), cis-cadin-4-en-7-ol (3.5%), caryophyllene oxide (3.2%), β-selinene (3.1%), α-humulene (2.8%), α-selinene (2.5%), selina-6-en-4β-ol (2.2%), selin-11-en-4α-ol (1.6%), hinesol (1.6%), humulene epoxide II (1.3%).	
M. leucadendra	Branch Tips	Broth microdilution	*C. albicans* (ATCC 10231	-	-	Nystatin (MIC): 8 μg/mL	α-Eudesmol (13.7%), *p* -cymene (8.7%), guaiol (7.3%), (E)-caryophyllene (5.7%), terpinolene (4.4%), β-selinene (4.2%), linalool (4.2%), α-selinene (4.1%), caryophyllene oxide (4%), α-humulene (3.7%), γ-terpinene (3.3%), eremoligenol (2.7%), bulnesol (2.2%), cis-cadin-4-en-7-ol (2.2%), γ-eudesmol (1.9%), humulene epoxide II (1.6%), γ-gurjunene (1.4%), α-pinene (1.4%), δ-selinene (1.3%), α-thujene (1.2%), selina-6-en-4β-ol (1.2%), α-amorphene (1.2%), *p* -cymen-8-ol (1.2%), terpinen-4-ol (1.1%), selin-11-en-4α-ol (1%).	
M. quinquenervia	Leaves	Broth microdilution	*C. albicans* (ATCC 10231)	1 mg/mL (1000 μg/mL)	-	Amphotericin B (MIC): 4 mg/mL (4000 μg/mL) Nystatin (MIC): 2 mg/mL (2000 μg/mL)	1,8-Cineole (40.3%), carveol (27.15%), myrtenol (9.43%), eucarvone (5.7%), camphor (5.66%), nopinone (1.9%), sabinene hydrate (1.7%).	[18]

	C. albicans	4 mg/mL (4000 μg/mL)	-	Amphotericin B (MIC): 4 mg/mL (4000 μg/mL) Nystatin (MIC): 2 mg/mL (2000 μg/mL)
	C. tropicalis (ATCC 750)	1 mg/mL (1000 μg/mL)	-	Amphotericin B (MIC): 4 mg/mL (4000 μg/mL) Nystatin (MIC): 2 mg/mL (2000 μg/mL)

Note: Diameter of inhibition zones includes diameter of disks (6 mm). * MIC: Minimum inhibitory concentration. ** MFC: Minimum fungicidal concentration. ATCC: American Type Culture Collection.

Table S2. Anti-*Candida* activity of *Citrus* spp. essential oils and their main components.

Citrus Species	Part of the Plant	Antifungal Activity					Major Components (>1%)	Reference
		**Method of Antifungal Susceptibility**	**Species of *Candida***	**Agar Diffusion or MIC ***	**MFC ****	**Comparison Drug Results**		
C. aurantifolia	-	Broth micro-dilution	*C. albicans* (ATCC 18804)	625 µL/mL	-	-	Limonene (51.9%), β-pinene (18.8%), γ-terpinene (8.1%).	[5]
C. aurantium	-	Broth micro-dilution	*C. albicans* (ATCC 18804)	625 µL/mL	-	-	Linalyl acetate (51.5%), linalool (25.4%).	
C. aurantium	Peel	Broth micro-dilution	*C. albicans* (SC 5314)	>2000 µg/mL	-	-	-	[16]
			C. glabrata (ATCC 2001)	>2000 µg/mL	-	-		
			C. krusei (ATCC 62580)	>2000 µg/mL	-	-		
			C. orthopsilosis (ATCC 96141)	>2000 µg/mL	-	-		
			C. parapsilosis (ATCC 22019)	>2000 µg/mL				
			C. tropicalis (ATCC 13803)	>2000 µg/mL	-	-		
C. aurantium	Young Leaf	Disk difusion	*C. albicans* (ATCC 90028)	25.3 ± 0.47 mm	-	Fluconazole (20 µg/disk): 18 ± 0.2 mm Amphotericin B (20 µg/disk): 21 ± 0.5 mm	2-βpinene (100%), δ-3 carene (84%), D-limonene (28%), β -myrcene (18.22%), citronella (11.6%), L-linalool (11.2%), γ -terpinene (9.08%), 3-cyclohexen-1-ol (8.74%), sabinene (5.93 %), citronellyl acetate (5.45 %).	[19]
			C. albicans (MTCC 277)	19 ± 0.02 mm	-	Fluconazole (20 µg/disk): 18 ± 0.2 mm Amphotericin B (20 µg/disk): 21 ± 0.5 mm		

C. aurantium	Young Leaf	Broth microdilution	*C. albicans* (ATCC 90028)	0.15% (*v*/*v*)	0.15% (*v*/*v*)	Fluconazole (MIC): 0.15% (*v*/*v*); (MFC): 0.15% (*v*/*v*) Amphotericin B (MIC): 0.62% (*v*/*v*); (MFC): 0.31% (*v*/*v*)	2-βpinene (100%), δ-3 carene (84%), D-limonene (28%), β -myrcene (18.22%), citronella (11.6%), L-linalool (11.2%), γ -terpinene (9.08), 3-cyclohexen-1-ol (8.74%), sabinene (5.93 %), citronellyl acetate (5.45 %).	[19]
			C. albicans (MTCC 277)	0.31% (*v*/*v*)	0.31% (*v*/*v*)	Fluconazole (MIC): 0.62% (*v*/*v*); (MFC): 0.62% (*v*/*v*) Amphotericin B (MIC): 0.62% (*v*/*v*); (MFC): 0.31% (*v*/*v*)		
C. bergamia	-	Broth microdilution	*C. albicans* (ATCC 18804)	625 µL/mL	-	-	Limonene (34.6%), linalyl acetate (34.3%), linalool (12.7%), γ -terpinene (6.6%), β-pinene (5.6%).	[5]
C. grandis	Leaves	Broth microdilution	*C. albicans* (ATCC 10231)	4 mg/ mL (4000 µg/mL)	-	Amphotericin B (20 µg/disk): 12.05 mm Nystatin (20 µg/disk): 12.05 mm	Borneol (42.24%), linalool (26.01%), linalyl acetate (19.89%), terpinen-4-ol (5.24%), fenchyl acetate (4.69%).	[18]
C. grandis	Peel	Broth microdilution	*C. albicans* (ATCC 10231)	4 mg/ mL (4000 µg/mL)	-	Amphotericin B: (MIC): 4 mg/mL (4000 µg/mL) Nystatin (MIC): 2 mg/mL (2000 µg/mL)	Limonene (75.43%), β -myrcene (3.52%), cycloisosativene (2.73%), terpinen-4-ol (2.35%), germacrene (2.09%), α -terpinene (2.05%), α -pinene (1.59%), β -pinene (1.57%).	[18]
			C. albicans	-	-	Amphotericin B (MIC): 4 mg/mL (4000 µg/mL) Nystatin (MIC): 2 mg/mL (2000 µg/mL)		

C. grandis	Peel	Broth microdilution	*C. tropicalis* (ATCC 750)	4 mg/ mL (4000 μg/mL)	-	Amphotericin B: (MIC): 4 mg/mL (4000 μg/mL) Nystatin (MIC): 2 mg/mL (2000 μg/mL)	Limonene (75.43%), β -myrcene (3.52%), cycloisosativene (2.73%), terpinen-4-ol (2.35%), germacrene (2.09%), α -terpinene (2.05%), α -pinene (1.59%), β -pinene (1.57%).	[18]
C. grandis	Leaves	Broth microdilution	*C. albicans* (ATCC 10231)	0.116 ± 0.004% (*v*/*v*)	-	-	Steam distillation (SD-EO): Citronellal (34.54%), citronellol (16.17%), citronellyl butyrate (10%), β-caryophyllene (8.22%), (−)-β-pinene (7.33%), trans-β-ocimene (6.8%), 3,7,11-tetramethyl bicyclo [8.1.0]2,6-undecadiene (3.43%), phytol (1.65%) e α-farnesene (1.57%), Sabinene (1.43%), dipentene (1.08%).	[20]
			C. albicans (ATCC 10231)	0.121 ± 0.007% (*v*/*v*)	-	-	Solvent-Free Microwave Extraction (SFME-EO): Citronellal (30.87%), citronellol (28.95%), (−)-β-pinene (9.58%), α-ocimene (6.38%), citronellyl butyrate (5.7%), Sabinene (3.01%), β-caryophyllene (2.76%), caryophyllene oxide (2.21%), dipentene (1.93%). spathulenol (1.02%).	[20]
C. hystix	Riped Fruits	Broth microdilution	*C. albicans* (ATCC 10231)	1 mg/mL (1000 μg/mL)	-	Amphotericin B: (MIC): 4 mg/mL (4000 μg/mL) Nystatin (MIC): 2 mg/mL (2000 μg/mL)	Limonene (83.89%), α-Pinene (3.02%).	[18]

			C. albicans	4 mg/mL (4000 μg/mL)	-	Amphotericin B: (MIC): 4 mg/mL (4000 μg/mL) Nystatin (MIC): 2 mg/mL (2000 μg/mL)		
			C. tropicalis (ATCC 750)	2 mg/mL (2000 μg/mL)	-	Amphotericin B: (MIC): 4 mg/mL (4000 μg/mL) Nystatin (MIC): 2 mg/mL (2000 μg/mL)		
C. latifolia	Peel	Agar difusion	*C. albicans*	0.4 μg/50 mL: 9.46 mm.	-	Amphotericin B (20 μg/disk): 12.05 mm	*d*-limonene (51.64%), β -thujene (14.85%), γ -terpinene (12.80%), β -pinene (12.79%), α -pinene (2.17%), α -myrcene (1.43%).	[21]
			C. glabrata	0.22 μg/50 mL: 8.52 mm.	-	Amphotericin B (20 μg/disk): 12.05 mm		
			C. guilliermondii	0.19 μg/50 mL: 8.94 mm.	-	Amphotericin B (20 μg/disk): 12.05 mm		
C. latifolia	Peel	Agar difusion	*C. lusitaniae*	1.09 μg/50 mL: 8.06 mm.	-	Amphotericin B (20 μg/disk): 12.05 mm	*d*-limonene (51.64%), β -thujene (14.85%), γ -terpinene (12.80%), β -pinene (12.79%), α -pinene (2.17%), α -myrcene (1.43%).	[21]
			C. tropicalis	1.3 μg/50 mL: 10.87 mm.	-	Amphotericin B (20 μg/disk): 12.05 mm		
C. limon	-	Broth micro-dilution	*C. albicans* (ATCC 18804)	625 μL/mL	-	-	Limonene (56.1%), β-pinene (15.8%), γ -terpinene (10.5%).	[5]
C. limon	Leaves	Broth micro-	*C. albicans* (SC 5314)	500 μg/mL	-	-	-	[16]

		dilution	*C. glabrata* (ATCC 2001)	250 μg/mL	-	-		
			C. krusei (ATCC 62580)	500 μg/mL	-	-		
			C. orthopsilosis (ATCC 96141)	500 μg/mL	-	-		
			C. parapsilosis (ATCC 22019)	500 μg/mL	-	-		
			C. tropicalis (ATCC 13803)	250 μg/mL				
C. limonum	-	Broth microdilution	*C. albicans* - Fluconazole Resistent	0.0097–0.312% (*v/v*)	0.0097–0.312% (*v/v*)	-	-	[12]
			C. glabrata- Fluconazole Resistent	0.0024–0.1565 (*v/v*)	0.0024–0.156% (*v/v*)	-		
			C. krusei- Fluconazole Resistent	0.0024–0.0097% (*v/v*)	0.0024–0.019% (v/v)	-		
C. limonum		Broth microdilution	*C. albicans*	<0.043 to >21.325 mg/mL	<0.043–>21.325 mg/mL	0.06 mg/mL	Citral (53.85%), others (27.18%), limonene (5.29%), terpinolene (4.57%), terpinene (4.49%).	[22]
			C. glabrata	<0.044 to 5.331 mg/mL	<0.043–5.331 mg/mL	-		
C. limonum	-	Cylinder-plate diffusion	*C. albicans*	(0.6–3.0%: 0 mm)	-	-	Limonene (48.27%), β-pinene (15.14%), α-pinene (11.06%), trans-citral (7.14%), c-terpinene (4.85%), cis-citral (4.3%), β-myrcene (4.11%), terpinolene (1.19%), geranyl acetate (0.91%).	[23]
			C. glabrata	(2.6%: 45 mm)<br>(3.0%: 44.8 mm)				
			C. tropicalis	(0.6–3.0%: 0 mm)	-	-		
C. limonum	-	Cylinder-plate diffusion	*C. albicans*	(0.6%: 44.8 mm)<br>(1.0%: 44.8 mm)<br>(1.6%: 44.8 mm)<br>(2.0%: 44.8 mm)<br>(2.6%: 45.0 mm)<br>(3.0%: 45.0 mm)	-	-	Limonene (23.39%), cis-citral (19.41%), trans-citral (15.52%), β-pinene (8.93%), α-caryophyllene (6.09%), c-terpinene (5.87%), cis-geraniol (4.96%), farnesol (4.48%), trans-geraniol (3.39%), β-myrcene (1.46%), α-pinene (1.44%).	

			C. glabrata	(0.6–3.0%: 0 mm)				
			C. tropicalis	(2.6%: 15.3 mm)<br>(3.0%: 16.3 mm)	-	-		
C. limonum	-	Cylinder-plate diffusion	*C. albicans*	(0.6%: 23 mm)<br>(1.0%: 25 mm)<br>(1.6%: 44.8 mm)<br>(2.0%: 44.8 mm)<br>(2.6%: 44.8 mm)<br>(3.0%: 45.0 mm)	-	-	Limonene (42.03%), β-pinene (15.15%), carvone (7.28%), 1-terpinen-4-ol (5.57%), pinocarveol (5.44%), verbenol (4.9%), α-pinene (3.42%), trans-carveol (3.36%), caryophyllene oxide (3.2%), α-bergamotene (2.31%), trans-p-2,8- (1.84%), trans-geraniol (1.58%), trans-verbenol (1.5%).	
			C. glabrata	(2.6%: 44.6 mm)<br>(3.0%: 44.8 mm)				
			C. tropicalis	(0.6–3.0%: 0 mm)	-	-		
C. limonum	-	Cylinder-plate diffusion	*C. albicans*	(3.0%: 17.4 mm)	-	-	Isopropyl myristate (42.78%), limonene (22.42%), bergamol (9.53%), β-ionene (7.81%), hydrocinnamic acid (6.54%), farnesol (6.12%), β-pinene (2.21%).	
			C. glabrata	(0.6–3.0%: 0 mm)				
			C. tropicalis	(0.6–3.0%: 0 mm)	-	-		
C. limonum	-	Cylinder-plate diffusion	*C. albicans*	(0.6–3.0%: 0 mm)	-	-	Limonene (63.2%), β-pinene (14.31%), β-myrcene (6.94%), c-terpinene (4.78%), α-pinene (3.61%), linalool (1.73%), citral (mix of isomers) (1.06%).	
			C. glabrata	(0.6–3.0%: 0 mm)				
			C. tropicalis	(0.6–3.0%: 0 mm)	-	-		
C. limonum	-	Cylinder-plate diffusion	*C. albicans*	(0.6–3.0%: 0 mm)	-	-	Limonene (38.5%), β-pinene (19.98%), β-felandrene (15.03%), α-pinene (5.56%), β-myrcene (5.42%), citral (mix of isomers) (2.63%), β-bisabolene (1.82%), cis-citral (1.54%), β-bergamotene (1.54%), Linalool (1.51%), 3-thujene (1.25%), neryl acetate (1.14%), caryophyllene (1.11%).	[23]
			C. glabrata	(0.6–3.0%: 0 mm)	-	-		
			C. tropicalis	(0.6–3.0%: 0 mm)				

C. nobilis	Peel	Broth microdilution	*C. albicans* (SC 5314)	2000 µg/mL	-	-		[16]
			C. glabrata (ATCC 2001)	2000 µg/mL	-	-		
			C. krusei (ATCC 62580)	>2000 µg/mL	-	-		
			C. orthopsilosis (ATCC 96141)	>2000 µg/mL	-	-		
			C. parapsilosis (ATCC 22019)	>2000 µg/mL				
			C. tropicalis (ATCC 13803)	>2000 µg/mL	-	-		
C. paradisi	-	Broth microdilution	*C. albicans* (ATCC 18804)	313 µL/mL	-	-	Limonene (91.3%).	[5]
C. paradisi	-	Broth microdilution	*C. albicans*	0.0039–1% (*v*/*v*)	-	Clotrimazole (MIC): >64 µg/mL Fluconazole (MIC): >64 µg/mL Itraconazole (MIC): >64 µg/mL	α-Copaene (0.1 %), α-pinen (0.55 %), β-caryophyllene (0.39 %), β-cubenene (0.09 %).	[9]
C. paradisi	-	Broth microdilution	*C. glabrata* (ATCC 15126)	0.007–1% (*v*/*v*)	-	Clotrimazole (MIC): 8 µg/mL Fluconazole (MIC): 8 µg/mL) Itraconazole (MIC): 1 µg/mL	-	[14]
C. paradisi	-	Broth microdilution	*C. glabrata*	-	-	Clotrimazole (MIC): 0.062 - 8 µg/mL Fluconazole (MIC): 2 - >64 µg/mL Itraconazole (MIC): 0.125 - >64 µg/mL	-	[14]

C. reticulata	-	Broth microdilution	*C. albicans* (ATCC 18804)	625 µL/mL	-	-	Limonene (91.3%).	[5]
C. reticulata	Peel	Broth microdilution	*C. albicans* (SC 5314)	2000 µg/mL	-	-	-	[16]
			C. glabrata (ATCC 2001)	1000 µg/mL	-	-		
			C. krusei (ATCC 62580)	250 µg/mL				
			C. orthopsilosis (ATCC 96141)	250 µg/mL				
			C. parapsilosis (ATCC 22019)	1000 µg/mL	-	-		
			C. tropicalis (ATCC 13803)	1000 µg/mL	-	-		
C. reticulata var. *Blanco*	Dry Leaves	Broth microdilution	*C. albicans* (SC 5314)	>2000 µg/mL	-	-	-	[16]
			C. glabrata (ATCC 2001)	2000 µg/mL	-	-		
			C. krusei (ATCC 62580)	>2000 µg/mL	-	-		
			C. orthopsilosis (ATCC 96141)	2000 µg/mL	-	-		
			C. parapsilosis (ATCC 22019)	2000 µg/mL	-	-		
			C. tropicalis (ATCC 13803)	>2000 µg/mL	-	-		
C. reticulata var. *Blanco*	Peels	Broth microdilution	*C. albicans* (SC 5314)	1000 µg/mL	-	-	-	[16]
			C. glabrata (ATCC 2001)	1000 µg/mL	-	-		
			C. krusei (ATCC 62580)	500 µg/mL	-	-		
			C. orthopsilosis (ATCC 96141)	1000 µg/mL	-	-		
			C. parapsilosis (ATCC 22019)	1000 µg/mL	-	-		

			C. tropicalis (ATCC 13803)	2000 µg/mL				
C. reticulata Blanco var *cravo*	Peels	Broth microdilution	*C. albicans* (SC 5314)	>2000 µg/mL	-	-	-	[16]
			C. glabrata (ATCC 2001)	2000 µg/mL	-	-		
			C. krusei (ATCC 62580)	>2000 µg/mL	-	-		
			C. orthopsilosis (ATCC 96141)	2000 µg/mL	-	-		
			C. parapsilosis (ATCC 22019)	>2000 µg/mL				
			C. tropicalis (ATCC 13803)	>2000 µg/mL	-	-		
C. reticulata Blanco var *cravo*	Fresh leaves	Broth microdilution	*C. albicans* (SC 5314)	2000 µg/mL	-	-	-	[16]
			C. glabrata (ATCC 2001)	>2000 µg/mL	-	-		
			C. krusei (ATCC 62580)	>2000 µg/mL	-	-		
			C. orthopsilosis (ATCC 96141)	>2000 µg/mL	-	-		
			C. parapsilosis (ATCC 22019)	>2000 µg/mL	-	-		
			C. tropicalis (ATCC 13803)	2000 µg/mL				
C. reticulata var *Blanco*	Riped Fruits	Broth microdilution	*C. albicans* (ATCC 10231)	0.3 mg/mL (300 µg/mL)	-	Amphotericin B (MIC): 4 mg/mL (4000 µg/mL) Nystatin (MIC): 2 mg/mL (2000 µg/mL)	Limonene (37.55%), β -pinene (13.24%), γ -terpinene (9.11%), β -myrcene (6.85%), terpinen-4-ol (6.32%), cymene (5.21%), α -pinene (4.7%), α -3-carene (3.11%), linoleic acid ethyl ester (2.44%), α -terpineol (1.05%).	[16]
			C. albicans	4 mg/mL (4000 µg/mL)	-	Amphotericin B (MIC): 4 mg/mL (4000 µg/mL) Nystatin (MIC): 2 mg/mL (2000 µg/mL)		
			C. tropicalis (ATCC 750)	2 mg/mL (2000 µg/mL)	-	Amphotericin B (MIC): 4 mg/mL (4000 µg/mL)		

						Nystatin (MIC): 2 mg/mL (2000 µg/mL)		
C. sinensis	Peel	Agar diffusion	*C. albicans*	1.68 µg/50 mL: 5.51 mm.	-	Amphotericin B (20 µg/disk): 12.05 mm	*d*-limonene (96.046%), α -myrcene (2.796%).	[21]
			C. glabrata	0.42 µg/50 mL: 5.78 mm.	-	Amphotericin B (20 µg/disk): 12.05 mm		
			C. guilliermondii	-	-	Amphotericin B (20 µg/disk): 12.05 mm		
			C. lusitaniae	3.71 µg/50 mL: 2.00 mm	-	Amphotericin B (20 µg/disk): 12.05 mm		
			C. tropicalis	0.72 µg/50 mL: 4.44 mm.	-	Amphotericin B (20 µg/disk): 12.05 mm		
C. sinensis	Peel	Broth microdilution	*C. albicans* (SC 5314)	>2000 µg/mL	-	-	-	[16]
			C. glabrata (ATCC 2001)	>2000 µg/mL	-	-		
			C. krusei (ATCC 62580)	>2000 µg/mL	-	-		
			C. orthopsilosis (ATCC 96141)	>2000 µg/mL	-	-		
			C. parapsilosis (ATCC 22019)	>2000 µg/mL	-	-		
			C. tropicalis (ATCC 13803)	>2000 µg/mL				

Note: Diameter of inhibition zones includes diameter of disks (6 mm). * MIC: Minimum inhibitory concentration. ** MFC: Minimum fungicidal concentration. ATCC: American Type Culture Collection.

Table S3. Anti-*Candida* activity *Cupressus* spp. essential oils and their main components.

Cupressus Species	Part of the Plant	Antifungal Activity					Major Components (>1%)	Reference
		Method of Antifungal Susceptibility	Species of *Candida*	Agar diffusion or MIC *	MFC **	Comparison Drug Results		
C. lusitanica	Aerial parts	Agar disk diffusion	*C. albicans* (ATCC 1098) Collection date: 17 January	7.5 ± 0.0 mm	-	Amphotericin B (10 µg/mL/disk): 13–15 ± 0.0 mm	Limonene (19.5%), sabinene (17.7%), δ-3-carene (16.8%), α-pinene (13.8%), β-myrcene (2.9%), terpinen-4-ol (2.3%), cadina-3,5-diene (2.3%), γ-terpinene (2%), terpinolene (2%), β-phellandrene (1.9%), α-terpinene (1.3%), trans-pinocarveol (1.3%), cis-thujopsene (1.1%), camphene (1%).	[24]
			C. albicans (ATCC 1098) Collection date: 17 February	8.5 ± 0.0 mm			α-Pinene (35.7%), sabinene (13.4%), limonene (7.7%), δ-3-carene (7.6%), β-phellandrene (4.6%), β-myrcene (4.4%), umbellulone (3.2%), terpinen-4-ol (2.9%), terpinolene (2.5%), γ-terpinene (2.3%), α-terpinene (2%), α-thujene (1.6%), cadina-3,5-diene (1.4%).	
C. lusitanica	Leaves -non-flowering	Agar disk diffusion	*C. albicans* (ATCC 9002)	100%, 10%, 1% (*v*/*v*): respectively, 13 mm, 7 mm, 6 mm	-	Nystatin (10 µg/disk): 22 mm	Germacrene D (18.5%), epi-zonarene (8.2%), cis-calamenene (8.2%), terpinen-4-ol (6.3%), linalool (6%), umbellulone (6%), di-epi-α-cedrene (4.9%), α-curcumene (4.1%), limonene (2.3%), α-amorphene (2%), β-caryophyllene (1.5%), linalyl acétate (1.2%), cedrol (1.2%), α-muurolene (1.1).	[25]
			C. glabrata (IP 35)	100%, 10%, 1% (*v*/*v*): respectively, 6 mm, 6 mm, 6 mm	-	Nystatin (10 µg/disk): 23 mm		
			C. krusei (ATCC 6258)	100%, 10%, 1% (*v*/*v*): respectively, 10 mm, 6 mm, 6 mm	-	Nystatin (10 µg/disk): 18 mm		

			C. lusitaniae (ATCC 200950)	100%, 10%, 1% (*v*/*v*): respectively, 13 mm, 10 mm, 6 mm	-	Nystatin (10 µg/disk): 33 mm		
			C. parapsilosis (ATCC 22019)	100%, 10%, 1% (*v*/*v*): respectively, 7 mm, 6mm, 6 mm	-	Nystatin (10 µg/disk): 24 mm		
			C. tropicalis (ATCC 750)	100%, 10%, 1% (*v*/*v*): respectively, 14 mm, 7 mm, 6 mm	-	Nystatin (10 µg/disk): 23 mm		
C. lusitanica	Leaves non-flowering	Macrowell dilution	*C. albicans* (ATCC 9002)	0.16% (*v*/*v*)	0.16% (*v*/*v*)	Nystatin (MIC): 2 µg/mL; (MFC): 2 µg/mL	Germacrene D (18.5%), epi-zonarene (8.2%), cis-calamenene (8.2%), terpinen-4-ol (6.3%), linalool (6%), umbellulone (6%), di-epi-α-cedrene (4.9%), α-curcumene (4.1%), limonene (2.3%), α-amorphene (2%), β-caryophyllene (1.5%), linalyl acétate (1.2%), cedrol (1.2%), α-muurolene (1.1%).	
			C. glabrata (IP 35)	1.25% (*v*/*v*)	1.25% (*v*/*v*)	Nystatin (MIC): 16 µg/mL; (MFC); 16 µg/mL		
			C. krusei (ATCC 6258)	1.25% (*v*/*v*)	1.25% (*v*/*v*)	Nystatin (MIC): 4 µg/mL; (MFC): 4 µg/mL		
			C. lusitaniae (ATCC 200950)	0.62% (*v*/*v*)	0.62% (*v*/*v*)	Nystatin (MIC): 2 µg/mL; (MFC): >2 µg/mL		
			C. parapsilosis (ATCC 22019)	1.25% (*v*/*v*v)	1.25% (*v*/*v*)	Nystatin (MIC): 16µg/mL; (MFC): 16µg/mL		
			C. tropicalis (ATCC 750)	1.25% (*v*/*v*)	1.25% (*v*/*v*)	Nystatin (MIC): 8 µg/mL; (MFC): 8 µg/mL		
C. arizonica var. *glabra*	Aerial parts	Broth mcrodilution	*C. albicans* (ATCC 18804)	5×10^{-2} (0.05 µL/mL))	-	-	α-Pinene (26.53%), umbellulone (15.05%), β -cubebene (6.71%), cal-	[26]

			C. dublinenensis (CIPO 82)	1×10^{-2} (0.01 µL/mL)	-	-	manene (4.5%), limonene (4.12%), terpinen-4-ol (4.08%), 14-norcadin-5-en-4-one (2.78%), 6-terpinene (2.06%), β -sesquiphellandrene (2.01%), camphor (1.83%), sabinene (1.7%), aromadendrene (1.65%), β -fenchol (1.48%), þ-cymene (1.47%), cedrol (1.36%), ionole (1.26%), 6-3-carene (1.02%).	
			C. glabrata (8D)	5×10^{-2} (0.05 µL/mL)	-	-		
			C. parapsilosis (28 B)	5×10^{-2} (0.05 µL/mL))	-	-		
			C. tropicalis (IGC 3097)	1×10^{-3} (0.001 µL/mL)	-	-		
C. arizonica var. *arizonica*	Aerial parts	Broth mcrodilution	*C. albicans* (ATCC 18804)	5 x 10^{-2} (0.05 µL/mL))	-	-	α-Pinene (29.76%), umbellulone (11.86%), terpinen-4-ol (5.72%), α-cedrene (4.12%), limonene (4.09%), camphene hydrate (3.82%), β -sesquiphellandrene (3.11%), 6-Terpinene (2.86%), camphor (2.68%), sabinene (2.51%), 6-3-Carene (1.72%), þ -cymene (1.56%), Labd-(13E)-8,15-diol (1.55%), β -fenchol (1.38%), α-terpinene (1.07%), delta-cadinene (1.04%), α-terpinolene (1.03%), cedrol (1.01%).	
			C. dublinenensis (CIPO 82)	1×10^{-2} (0.01 µL/mL)	-	-		
			C. glabrata (8D)	1×10^{-2} (0.01 µL/mL)	-	-		
			C. parapsilosis (28 B)	1×10^{-2} (0.01 µL/mL)	-	-		
			C. tropicalis (IGC 3097)	1×10^{-2} (0.01 µL/mL)	-	-		
C. semper-virens	Aerial parts - flowering period	Broth microdilution	*C. albicans* (ATCC 10231)	0.42 ± 0.027 µL/mL	1.319 ± 0.066 µL/mL	Nystatin (MIC_{90}): 0.84 ± 0.062; (MFC): 1 ± 0.062	(+)-Cedrol (74.03%), α-pinene (4.6%), thymol (4.25%), δ-3-carene (3.8%), (-)-caryophyllene oxide (3.31%), (-)-borneol (2.33%), α-cadinol (2.19%).	[27]
			C. glabrata	<64 µL/mL	-	Nystatin (MIC_{90}): 0.84 ± 0.032; (MFC): 1 ± 0.045		
			C. krusei	<64 µL/mL	-	Nystatin (MIC_{90}): 1.148 ± 0.065; (MFC): 2 ± 0.034		

			C. parapsilosis	0.757 ± 0.067 µL/mL	1.64 ± 0.058 µL/mL	Nystatin (MIC_{90}/MFC): 0.84 ± 0.076 /1 ± 0.041		
C. semper-vires	Leaves	Broth microdilution	*C. albicans* (SC 5314)	250 µg/mL	-	-	Sabinene (20.3%), citral (20%), ter-pinen-4-ol (15.4%), α-pinene (8%), myrcene (6%), þ -cymene (5%), neral (5%), δ-2-carene (4%), γ-terpinene (4%), limonene (3.9%), δ-cadinene (3%), α-terpineol (2.4%), cedrol (2.1%).	[16]
			C. glabrata (ATCC 2001)	31.25 µg/mL	-	-		
			C. krusei (ATCC 62580)	62.5 µg/mL	-	-		
			C. orthopsilosis (ATCC 93141)	31.25 µg/mL	-	-		
			C. parapsilosis (ATCC 22019)	62.5 µg/mL	-	-		
			C. tropicalis (ATCC 13803)	250 µg/mL	-	-		
C. semper-virens	-	Broth microdilution	*C. albicans* (ATCC 18804)	625 µg/mL	-	-	α-Pinene (49.7%), δ-3-carene (27.0%).	[5]
C. macro-carpa	Leaves	Broth microdilution	*C. albicans* (ATCC 10231)	1 mg/mL (1000 µg/mL)	40% of all MFCs were no longer than a higher dilution.	Amphotericin B (MIC): 4 mg/mL (4000 µg/mL) Nystatin (MIC): 1,5 mg/mL (1500 µg/mL)	α -Pinene (63.2%), cedrol (7.21%), β -myrcene (4.2%), β -pinene (4.1%), γ -terpinene (3.1%), germacrene (1.9%), sabinene (1.56%).	[18]
			C. albicans	2 mg/mL (2000 µg/mL)		Nystatin (MIC): 4 mg/mL (4000 µg/mL)		
			C. tropicalis (ATCC 750)	2 mg/mL (2000 µg/mL)		Amphotericin B (MIC): 4 mg/mL (4000 µg/mL) Nystatin (MIC): 2 mg/mL (2000 µg/mL)		

Note: Diameter of inhibition zones includes diameter of disks (6 mm). * MIC: Minimum inhibitory concentration. ** MFC: Minimum fungicidal concentration. ATCC: American Type Culture Collection .

Table S4. Anti-*Candida* activity of *Litsea* spp. essential oils and their main components.

Litsea Species	Part of the Plant	Antifungal Activity					Major Components (>1%)	Reference
		**Method of Antifungal Susceptibility**	**Species of *Candida***	**MIC ***	**MFC ****	**Comparison Drug Results**		
L. viridis	Leaf	Broth microdilution	*C. albicans* (ATCC 10231)	128 μg/mL	-	Nystatin (MIC): 8 μg/mL	Bicyclogermacrene (25.5%), decanal (14.4%), α-pinene (11.1%), β-pinene (8.3%), aromadendrene (3%), do-decanal (2%), β-elemene (1.9%), limonene (1.8%), methyl (E)-cinnamate (1.5%), germacrene B (1.3%), β-selinene (1.2%), (E)-nerolidol (1.1%), γ-elemene (1%), germacrene D (1%), (E)-γ-bisabolene (1%).	[28]
L. cubeba	Fruits	Broth microdilution	*C. albicans* (SC 5314)	500 μg/mL	-	-	Limonene (37%), neral (31.4%), citral (12%), linalool (4%), α-terpineol (2.3%), α-pinene (2%), β-pinene (2%), β-caryophyllene (1.7%), sabinene (1.3%), myrcene (1.3%), geraniol (1.2%).	[16]
			C. tropicalis (ATCC 13803)	1000 μg/mL	-	-		
			C. krusei (ATCC 62580)	62.5 μg/mL	-	Amphotericin B (MIC): 1.00 μg/mL		
			C. glabrata (ATCC 2001)	250 μg/mL	-	-		
			C. parapsilosis (ATCC 22019)	500 μg/mL	-	Amphotericin B (MIC): 0.25 μg/mL		
			C. parapsilosis (ATCC 96141)	250 μg/mL	-	-		

Note: * MIC: Minimum inhibitory concentration. ** MFC: Minimum fungicidal concentration.

References

1. Ramadan, M.A.; Shawkey, A.E.; Rabeh, M.A.; Abdellatif, A.O. Promising antimicrobial activities of oil and silver nanoparticles obtained from *Melaleuca alternifolia* leaves against selected skin-infecting pathogens. *J. Herb. Med*. **2020**, *20*, 100289.
2. Noumi, E.; Snoussi, M.; Hajlaoui, H.; Trabelsi, N.; Ksouri, R.; Valentin, E.; Bakhrouf, A. Chemical composition, antioxidant and antifungal potential of *Melaleuca alternifolia* (tea tree) and *Eucalyptus globulus* essential oils against oral *Candida* species. *J. Med. Plants Res*. **2011**, *5*, 4147–4156.
3. Antinori, S.; Milazzo, L.; Sollima, S.; Galli, M.; Corbellino, M. Candidemia and invasive candidiasis in adults: A narrative review. *Eur. J. Intern. Medicine*. **2016**, *34*, 21–28.
4. Rajkowska, K.; Kunicka-Styczyńska, A.; Maroszyńska, M. Selected Essential Oils as Antifungal Agents Against Antibiotic-Resistant *Candida* spp.: In Vitro Study on Clinical and Food-Borne Isolates. *Microb. Drug Resist*. **2017**, *23*, 18–24.
5. Powers, C.N.; Osier, J.L.; McFeeters, R.L.; Brazell, C.B.; Olsen, E.L.; Moriarity, D.M. Antifungal and Cytotoxic Activities of Sixty Commercially-Available Essential Oils. *Molecules* **2018**, *23*, 1549.
6. Serra, E.; Hidalgo-Bastida, L.A.; Verran, J.; Williams, D.; Malic, S. Antifungal Activity of Commercial Essential Oils and Biocides against *Candida albicans*. *Pathogens* **2018**, *7*, 15.
7. Rajkowska, K.; Nowak, A.; Kunicka-Styczyńska, A.; Siadura, A. Biological effects of various chemically characterized essential oils: Investigation of the mode of action against *Candida* albicans and HeLa cells. *RSC Adv*. **2016**, *6*, 97199–97207.
8. de Campos-Rasteiro, V.M.; da Costa, A.C.B.P.; Araújo, C.F.; Barros, P.P.; Rossoni, R.D.; Anbinder, A.L.; Jorge, A.O.C.; Junqueira, J.C. Essential oil of *Melaleuca alternifolia* for the treatment of oral candidiasis induced in an immunosuppressed mouse model. *BMC Complement. Altern. Med*. **2014**, *14*, 489.
9. Bona, E.; Cantamessa, S.; Pavan, M.; Novello, G.; Massa, N.; Rocchetti, A.; Berta, G.; Gamalero, E. Sensitivity of *Candida albicans* to essential oils: are they an alternative to antifungal agents? *J. Appl. Microbiol.* **2016**, *121*, 1530–1545.
10. Ramage, G.; Milligan, S.; Lappin, D.F.; Sherry, L.; Sweeney, P.; Williams, C.; Bagg, J.; Culshaw, S. Antifungal, Cytotoxic, and Immunomodulatory Properties of Tea Tree Oil and Its Derivative Components: Potential Role in Management of Oral Candidosis in Cancer Patients. *Front. Microbiol.* **2012**, *3*, 220.
11. Ninomiya, K.; Maruyama, N.; Inoue, S.; Ishibashi, H.; Takizawa, T.; Oshima, H.; Abe, S. The Essential Oil of Melaleuca alternifolia (Tea Tree Oil) and Its Main Component, Terpinen-4-ol Protect Mice from Experimental Oral Candidiasis. *Biol. Pharm. Bull.* **2012**, *35*, 861–865.
12. Szweda, P.; Gucwa, K.; Kurzyk, E.; Romanowska, E.; Dzierżanowska-Fangrat, K.; Zielińska-Jurek, A. Essential Oils, Silver Nanoparticles and Propolis as Alternative Agents Against Fluconazole Resistant *Candida albicans*, *Candida glabrata* and *Candida krusei* Clinical Isolates. *Indian J. Microbiol*. **2015**, *55*, 175–183.
13. Brun, P.; Bernabè, G.; Filippini, R.; Piovan, A. In Vitro Antimicrobial Activities of Commercially Available Tea Tree (*Melaleuca alternifolia*) Essential Oils. *Curr. Microbiol*. **2019**, *76*, 108–116.
14. Massa, N.; Cantamessa, S.; Novello, G.; Ranzato, E.; Martinotti, S.; Pavan, M.; Rocchetti, A.; Berta, G.; Gamalero, E.; Bona, E. Antifungal activity of essential oils against azole-resistant and azole-susceptible vaginal *Candida glabrata* strains. *Can. J. Microbiol.* **2018**, *64*, 647–663.
15. Tullio, V.; Roana, J.; Scalas, D.; Mandras, N. Enhanced Killing of *Candida krusei* by Polymorphonuclear Leucocytes in the Presence of Subinhibitory Concentrations of *Melaleuca alternifolia* and “Mentha of Pancalieri” Essential Oils. *Molecules* **2019**, *24*, 3824.
16. Pedroso, R.D.S.; Balbino, B.L.; Andrade, G.; Dias, M.C.P.S.; Alvarenga, T.A.; Pedroso, R.C.N.; Pimenta, L.P.; Lucarini, R.; Pauletti, P.M.; Januário, A.H.; et al. In Vitro and In Vivo Anti-*Candida* spp. Activity of Plant-Derived Products. *Plants* **2019**, *8*, 494.
17. Ramadan, M.A.; Shawkey, A.E.; Rabeh, M.A.; Abdellatif, A.O. Promising antimicrobial activities of oil and silver nanoparticles obtained from *Melaleuca alternifolia* leaves against selected skin-infecting pathogens. *J. Herb. Med*. **2020**, *20*, 100289.
18. Aumeeruddy-Elalfi, Z.; Gurib-Fakim, A.; Mahomoodally, F. Antimicrobial, antibiotic potentiating activity and phytochemical profile of essential oils from exotic and endemic medicinal plants of Mauritius. *Ind. Crop. Prod*. **2015**, *71*, 197–204.
19. Nidhi, P.; Rolta, R.; Kumar, V.; Dev, K.; Sourirajan, A. Synergistic potential of *Citrus aurantium* L. essential oil with antibiotics against *Candida albicans*. *J. of Ethnopharmacol*. **2020**, *262*, 113135.
20. Tsai, M.-L.; Lin, C.-D.; Khoo, K.A.; Wang, M.-Y.; Kuan, T.-K.; Lin, W.-C.; Zhang, Y.-N.; Wang, Y.-Y. Composition and Bioactivity of Essential Oil from *Citrus grandis* (L.) Osbeck “Mato Peiyu” Leaf. *Molecules.* **2017**, *22*, 2154.
21. Ruiz-Pérez, N.J.; González-Ávila, M.; Sánchez-Navarrete, J.; Toscano-Garibay, J.D.; Moreno-Eutimio, M.A.; Sandoval-Hernández, T.; Arriaga-Alba, M. Antimycotic Activity and Genotoxic Evaluation of *Citrus sinensis* and *Citrus latifolia* Essential Oils. *Sci. Rep*. **2016**, *6*, 253–271.

22. Gucwa, K.; Milewski, S.; Dymerski, T.; Szweda, P. Investigation of the Antifungal Activity and Mode of Action of *Thymus vulgaris*, *Citrus limonum*, *Pelargonium graveolens*, *Cinnamomum cassia*, *Ocimum basilicum*, and *Eugenia caryophyllus* Essential Oils. *Molecules* **2018**, *23*, 1116.
23. Białoń, M.; Krzyśko-Łupicka, T.; Koszałkowska, M.; Wieczorek, P.P. The Influence of Chemical Composition of Commercial Lemon Essential Oils on the Growth of *Candida* Strains. *Mycopathologia* **2014**, *177*, 29–39.
24. Tavares, C.S.; Martins, A.; Faleiro, M.L.; Miguel, M.G.; Duarte, L.C.; Gameiro, J.A. Bioproducts from forest biomass: Essential oils and hydrolates from wastes of *Cupressus lusitanica* Mill. and *Cistus ladanifer* L. *Industrial Crops and Products*. **2020**, *144*, 112034.
25. Teke, G.N.; Elisée, K.N.; Roger, K.J. Chemical composition, antimicrobial properties and toxicity evaluation of the essential oil of *Cupressus lusitanica* Mill. leaves from Cameroon. *BMC Complement. Altern. Med*. **2013**, *13*, 130.
26. Khouaja, W.; Oliveira, R.; Raies, A.; Dias, A.C.P. Antifungal activity of the essential oils from *Cupressus arizonica* var. *arizonica* and var. *glabra*. *Ind. Crop. Prod.* **2015**, *77*, 614–623.
27. Ibrahim, T.A.; El-Hela, A.A.; El-Hefnawy, H.M.; Al-Taweel, A.M.; Perveen, S. Chemical Composition and Antimicrobial Activities of Essential Oils of Some Coniferous Plants Cultivated in Egypt. *Iran. J. Pharm. Res.* **2017**, *16*, 328–337.
28. Chau, D.T.M.; Chung, N.T.; Huong, L.T.; Hung, N.H.; Ogunwande, I.A.; Dai, D.N.; Setzer, W.N. Chemical Compositions, Mosquito Larvicidal and Antimicrobial Activities of Leaf Essential Oils of Eleven Species of Lauraceae from Vietnam. *Plants* **2020**, *9*, 606.
